# Accelerating syngas-to-aromatic conversion via spontaneously monodispersed Fe in ZnCr_2_O_4_ spinel

**DOI:** 10.1038/s41467-022-33217-9

**Published:** 2022-09-22

**Authors:** Guo Tian, Xinyan Liu, Chenxi Zhang, Xiaoyu Fan, Hao Xiong, Xiao Chen, Zhengwen Li, Binhang Yan, Lan Zhang, Ning Wang, Hong-Jie Peng, Fei Wei

**Affiliations:** 1 Beijing Key Laboratory of Green Chemical Reaction Engineering and Technology, Department of Chemical Engineering, Tsinghua University, Beijing, 100084 China; 2 Institute of Fundamental and Frontier Sciences, University of Electronic Science and Technology of China, Chengdu, 611731 Sichuan China; 3 Faculty of Environment and Life, Beijing University of Technology, Beijing, 100124 China

**Keywords:** Heterogeneous catalysis, Chemical engineering, Catalyst synthesis

## Abstract

Spontaneous monodispersion of reducible active species (e.g., Fe, Co) and their stabilization in reductive atmospheres remain a key challenge in catalytic syngas chemistry. In this study, we present a series of catalysts including spontaneously monodispersed and enriched Fe on ZnCr_2_O_4_. Deep investigation shows remarkable performance in the syngas-to-aromatic reaction only when monodispersed Fe coupled with a H-ZSM-5 zeolite. Monodispersed Fe increases the turnover frequency from 0.14 to 0.48 s^−1^ without sacrificing the record high selectivity of total aromatics (80–90%) at a single pass. The increased activity is ascribed to more efficient activation of CO and H_2_ at oxygen vacancy nearest to the isolated Fe site and the prevention of carbide formation. Atomic precise characterization and theoretical calculations shed light on the origin and implications of spontaneous Fe monodispersion, which provide guidance to the design of next-generation catalyst for upgrading small molecules to synthetic fuels and chemicals.

## Abstract (web summary)

Spontaneous monodispersion of active species and their stabilization in reductive atmospheres remain a challenge in catalytic syngas chemistry. Here the authors demonstrate that syngas-to-aromatic conversion can be significantly accelerated by the spontaneously monodispersed Fe in ZnCr_2_O_4_ spinel.

## Introduction

The spontaneous dispersion of catalytically active species on the support has been widely investigated in heterogeneous catalysis due to interactions between the species and support^1,2^. Highly dispersed or, more strictly, monodispersed active sites endow the supported catalysts with unique catalytic properties. Spontaneous monodispersion can also improve atomic efficiency. Metal oxides with weak internal cohesive energy, e.g., MoO_3_ and NiO, can be easily dispersed on supports such as γ-Al_2_O_3_ and TiO_2_, manifesting as prototype designs of spontaneously dispersed active sites^2–4^. Such spontaneous dispersion is driven by negative Gibbs free energy (Δ*G* < 0) of breaking internal bonds of oxides being dispersed and forming new bonds between dispersed species and support. From this thermodynamics perspective, the monodispersion of species with strong self-interaction, such as Fe, Co, and Pt, presents a daunting challenge in heterogeneous catalysis.

Confining Fe, Co, and Pt precursors into the cages of nanoporous materials is an effective means of preparing monodispersed catalysts for many heterogeneous catalytic processes^5^. However, owing to the high surface energy, monodispersed Fe, Co, and Pt species tend to migrate and agglomerate under reaction conditions, especially at elevated temperatures, which inevitably cause catalyst deactivation. For instance, Han et al.^6^ found that the formation of Fe nanoclusters resulted in the deactivation of monodispersed Fe–N–C catalysts in proton exchange membrane fuel cells. Improving the metal–support interaction, using oxygen-donor support was considered as an efficient method to stabilize monodispersed species^7^. Recently, Nie et al.^8^ demonstrated how atomically dispersed ionic Pt^2+^ was stabilized by CeO_2_ to endure steam treatment, presenting a feasible way against catalyst sintering. In addition to agglomeration, reductive atmosphere such as syngas (CO/H_2_) can induce irreversible phase transition from metals to metal carbides, e.g., Fe to Fe_*x*_C_*y*_, Co to Co_x_C_y_, and the as-generated carbides further complicate the reaction pathways of syngas conversion and result in unwanted products^9–11^. Therefore, to stabilize monodispersed Fe and Co species and to avoid Fe_*x*_C_*y*_, Co_x_C_y_ formation in a reactive atmosphere have become a major bottleneck in catalytic syngas conversion.

In this study, we rationalized ZnCr_2_O_4_ spinel with abundant oxygen vacancies (O_V_) as oxygen-donor support. Fe could be spontaneously monodispersed on ZnCr_2_O_4_ via a simple impregnation method when the content of Fe was controlled to less than 5 wt%. The octahedral site preference energies from the literature^12^ are in the order of Fe^3+^ < Fe^2+^ < Cr^3+^ < Zn^2+^, indicating that Fe^2+^ or Fe^3+^ prefers to occupy the octahedral sites, whereas Zn^2+^ tends to occupy the tetrahedral sites in the spinel structure. The spontaneously monodispersed Fe was found not only thermodynamically stable at high temperature but also resistive to carbonization in a reducing atmosphere. When coupled with an H-ZSM-5 zeolite to form a composite catalyst, ZnCr_2_O_4_ with monodispersed Fe showed significantly improved syngas conversion efficiency (44%) with a turnover frequency (TOF) increased from 0.14 to 0.48 s^−1^; while the high selectivity towards aromatics was maintained by preventing the formation of Fe_*x*_C_*y*_. Moreover, the composite catalyst exhibited stable performance for more than 100 h reaction at 350 °C and 2.0 MPa. Density functional theory (DFT) calculations revealed that monodispersed Fe sites in ZrCr_2_O_4_ greatly reduced energy barriers of forming formaldehyde (H_2_CO) and methoxy (CH_3_O*) from syngas, which were previously identified as key intermediates leading to aromatics^11,13–15^. This study not only demonstrates a promising monodispersed catalyst that is highly active, selective, and stable in a reactive syngas atmosphere but also sheds light on spontaneous monodispersion of catalytically active species for heterogeneous catalysis.

## Results and discussion

### Identification of spontaneously monodispersed and enriched Fe

**Fig. 1:**
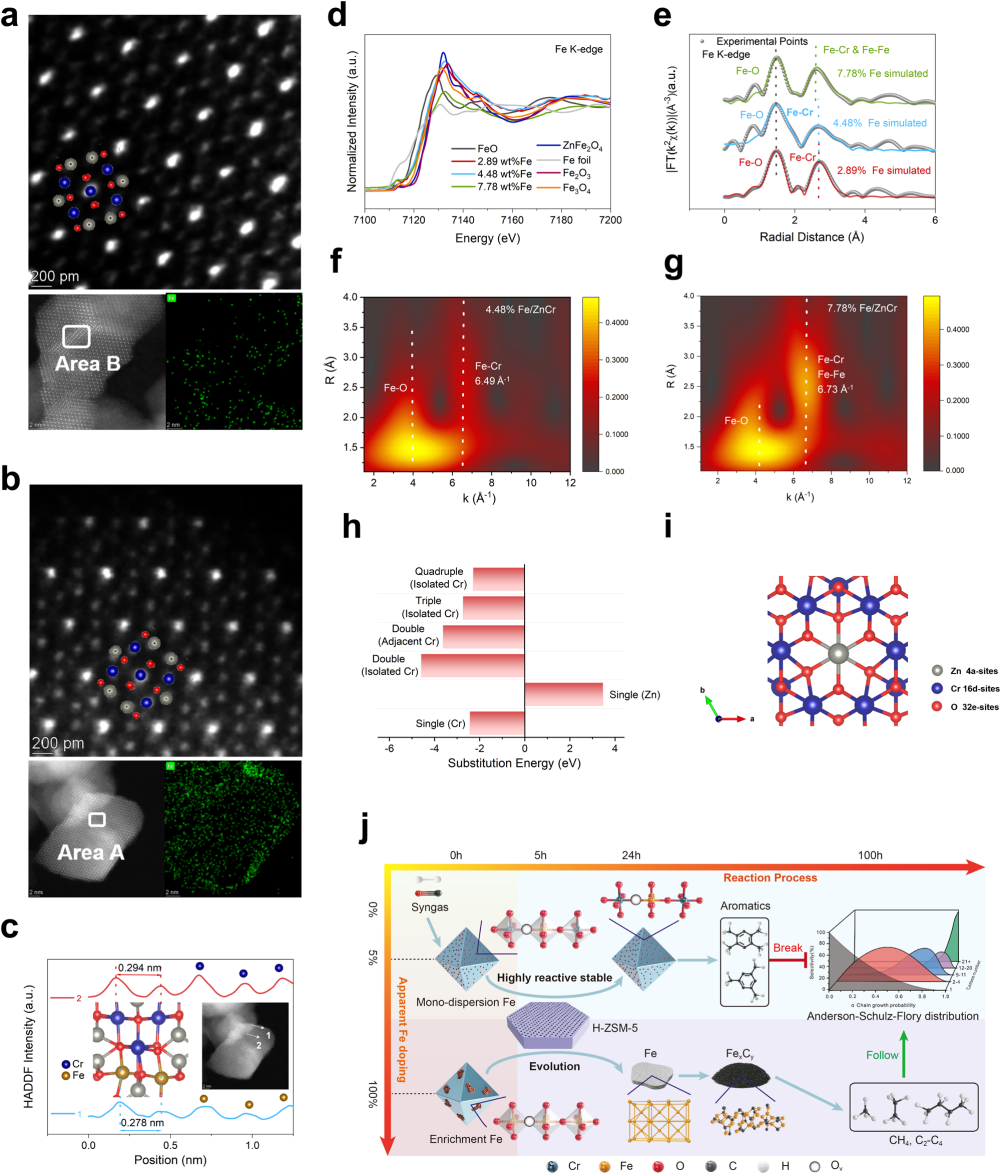
Fe monodispersion on ZnCr_2_O_4_ spinel. HADDF-STEM images of **a** 4.48% Fe-Zn/Cr and **b** 7.78% Fe-Zn/Cr and corresponding EDS mappings of Fe element. **c** HADDF intensity profile of two lines at the selected regions shown in the inset HADDF-STEM image. The atomic structure of Zn(Cr, Fe)_2_O_4_ spinel is also shown as an inset to illustrate the distance between two adjacent (16d) sites. **d** Fe K-edge X-ray absorption near-edge structure (XANES) profiles of different Fe-Zn/Cr and some standard samples. **e** The first two shells, Fe–O and Fe–Cr/Fe–Fe fittings, of the Fe K-edge Fourier transform-extended X-ray absorption fine structure (FT-EXAFS) spectra of different Fe-Zn/Cr. **f**, **g** Fe K-edge wavelet transform (WT)-EXAFS spectra of 4.48% Fe-Zn/Cr and 7.78% Fe-Zn/Cr, respectively. **h** DFT calculated substitution energies of Fe in the 4a site and 16d sites (including isolated, adjacent, and multiple sites) of the ZnCr_2_O_4_ matrix. **i** Structure model of pristine ZnCr_2_O_4_ showing different sites. **j** Schematic illustration showing how monodispersed Fe endures reactive atmosphere and enriched Fe suffer from phase transition to Fe_*x*_C_*y*_ during syngas conversion.

ZnCr_2_O_4_ spinel with increasing amounts of Fe (denoted as X% Fe-Zn/Cr where X refers to the weight percentage of Fe) were prepared for investigation (Supplementary Fig. 1). The identical crystal structure of ZnFe_2_O_4_ and ZnCr_2_O_4_, as well as the similar atomic weights of Fe and Cr, makes it very challenging to identify the state of Fe in ZnCr_2_O_4_^16,17^. High-angle annular dark-field scanning transmission electron microscopy (HADDF-STEM) images of different Fe-Zn/Cr all show well-identified atomic lattices of the spinel [001] and [220] surfaces (Fig. 1a, b and Supplementary Figs. 2, 3). Nevertheless, the Fe, Cr, and Zn atoms cannot be distinguished only by the contrast of images. To probe the element distribution, HADDF energy dispersive spectroscopy (EDS) mapping at the atomic scale was employed. Elemental composition through EDS analysis agrees well with the quantitative inductively coupled plasma optical emission spectroscopy results (Supplementary Fig. 4). When the amount of Fe is less than 4.48 wt%, the distribution of Fe is uniform across the catalyst surface. In contrast, Fe is enriched at the catalyst edges when its amount further increases to 7.78 wt%. Furthermore, the spinel structure also exists at the edges, indicating that the Fe-enriched region might be assigned to ZnFe_2_O_4_ (Supplementary Fig. 5). This is further verified by DFT simulation of the [220] surfaces of ZnCr_2_O_4_ and ZnFe_2_O_4_, which show that the Cr (16d)–Cr (16d) distance is ~5–6% longer than the Fe (16d)–Fe (16d) distance (16d refers to an octahedral site; Supplementary Fig. 6). Such a difference aligns with the analysis of HADDF intensity profiles of a Fe-enriched region (arrow 1) and a Fe-deficient region (arrow 2), respectively (Fig. 1c). Distances between two adjacent 16d sites in the two regions are 0.278 nm and 0.294 nm, respectively. Therefore, it is inferred that when the Fe amount <4.48 wt%, it is feasible to monodisperse Fe on the ZnCr_2_O_4_ matrix spontaneously; when the Fe amount increases to 7.78 wt%, Fe starts to accumulate at the edge of the catalyst surface, likely in the form of ZnFe_2_O_4_.

Since electron microscopy analysis only reveals local structure information, XANES and EXAFS based on synchrotron radiation were performed to unveil the coordination structures more globally. The Fe XANES edges of different Fe-Zn/Cr is between those of FeO and Fe_2_O_3_, indicating that the average valence state of Fe is between +2 and +3 (Fig. 1d and Supplementary Fig. 7). FT-EXAFS spectra of different Fe-Zn/Cr reveals a Fe–O distance without phase correction (1.62 Å) similar to that of ZnFe_2_O_4_ (Fig. 1e and Supplementary Fig. 8a). While for the peak at around 2.8 Å, Fe-Zn/Cr samples with a Fe content ≤4.48 wt% show a bond length closer to Fe_3_O_4_ and 7.78% Fe-Zn/Cr is closer to ZnFe_2_O_4_. Although FT-EXAFS cannot distinguish the separate peaks with an atomic number of ±2, e.g., Fe–Fe and Fe–Cr, such a difference is coincided with the DFT calculations (Supplementary Fig. 6) and HADDF-STEM observation (Fig. 1c), suggesting that only the Fe–O–Cr structure exists in spontaneously monodispersed Fe samples (<4.48 wt%), whereas the Fe–O–Cr and Fe–O–Fe structures coexist in the enriched Fe sample (7.78 wt%). Further Fe 2p X-ray photoelectron spectroscopy (XPS) analysis shown in Supplementary Fig. 8b supports the above deduction from FT-EXAFS^18,19^.

The subtle difference in local coordination structures between samples with monodispersed and enriched Fe is further confirmed using WT-EXAFS (Fig. 1f, g and Supplementary Fig. 9). In general, the WT-EXAFS heat maps show two scattering centers, which are associated with Fe–O and Fe–Cr/Fe–Fe or Fe–Fe, respectively. The k-axis location of the second scattering center shifts to higher values with increasing Fe amounts (6.46, 6.49, 6.73, and 6.83 Å^−1^ when the Fe amount is 2.89, 4.48, 7.78, and 100 wt% (i.e., ZnFe_2_O_4_)), corresponding to shortened bond lengths (Supplementary Fig. 10). This aligns well with the transition from longer Fe–Cr to shorter Fe–Fe. The FT-EXAFS and WT-EXAFS results were further fitted using doped Fe-Zn/Cr systems after geometry optimization (Fig. 1e and Supplementary Fig. 11). Fitted results show that the coordination numbers (CNs) of Fe–O (i.e. the nearest neighboring shell) are more than 4 for all Fe-Zn/Cr samples, verifying the 16d site as Fe-substitution site because 4a site’s CN is less than 4 (Supplementary Table 1)^20^. All the fitting results, including the CN, bond distances (R), and Debye–Waller factor (σ^2^) are listed in Supplementary Table 1.

The above electron microscopic and synchrotron radiation-based spectroscopic characterizations provide experimental validation to the monodispersion of Fe when the Fe content is below 4.48 wt%. We then performed DFT calculations to understand the origin of Fe monodispersion. Energies that are required for Fe substituting Cr or Zn atoms at different sites on the (111) surface of ZnCr_2_O_4_ are shown in Fig. 1h, along with the atomic surface model of ZnCr_2_O_4_ (111) surface shown in Fig. 1i (calculation details are presented in Supplementary Information). Notably, the substitution of Cr by Fe at the (16d) site is thermodynamically preferred (−2.43 eV) over substitution of Zn at the (4a) site (3.47 eV) (4a refers to a tetrahedral site and all the substitution sites are shown in Supplementary Fig. 12a, b). When more Cr (16d) sites are considered for Fe substitution, it is found that Fe prefers to substitute a (16d) site far from the existing doping site rather than an adjacent one, with substitution energy being 0.96 eV more negative (Supplementary Fig. 12c, d). Namely, isolated (16d) sites are preferably occupied upon Fe doping. Note that calculated substitution energies at isolated (16d) sites remain negative when the doping concentration of Fe increases from 6.25 to 25% (normalized to all the surface metal atoms of spinel (111) surface), providing theoretical insights into spontaneous monodispersion of Fe on ZnCr_2_O_4_. By using DFT calculation, we also investigated the stability of the isolated Fe doping site in a reactive syngas condition. The formation energies of Fe_5_C_2_ (as a representative Fe_*x*_C_*y*_ phase) from Fe in ZnFe_2_O_4_ and monodispersed Fe in ZnCr_2_O_4_ are −3.17 eV and 1.25 eV per Fe atom, respectively, indicating the strong tendency of ZnFe_2_O_4_ and high resistance of Fe-Zn/Cr to form carbides (Supplementary Table 2). Therefore, we propose a catalyst evolution scene herein (Fig. 1j). In an oxide-zeolite (OX-ZEO) tandem catalytic system for syngas conversion, the oxide catalyzes the formation of oxygenates such as H_2_CO, CH_3_O* and CH_3_OH from syngas and the zeolite further selectively catalyzes these oxygenates to produce aromatics^21–23^. The Fe-enriched oxide catalysts, however, suffers from irreversible phase transition to Fe_*x*_C_*y*_, which follows Anderson–Schulz–Flory theory and inevitably leads to alkanes with a broad distribution of carbon atoms per product molecule. In contrast, the monodispersed Fe with superior stability against sintering and carbonization could enable stable and selective aromatics formation from syngas.

### Catalyst performance of monodispersed and enriched Fe in ZnCr_2_O_4_ coupled with H-ZSM-5 for syngas conversion

**Fig. 2:**
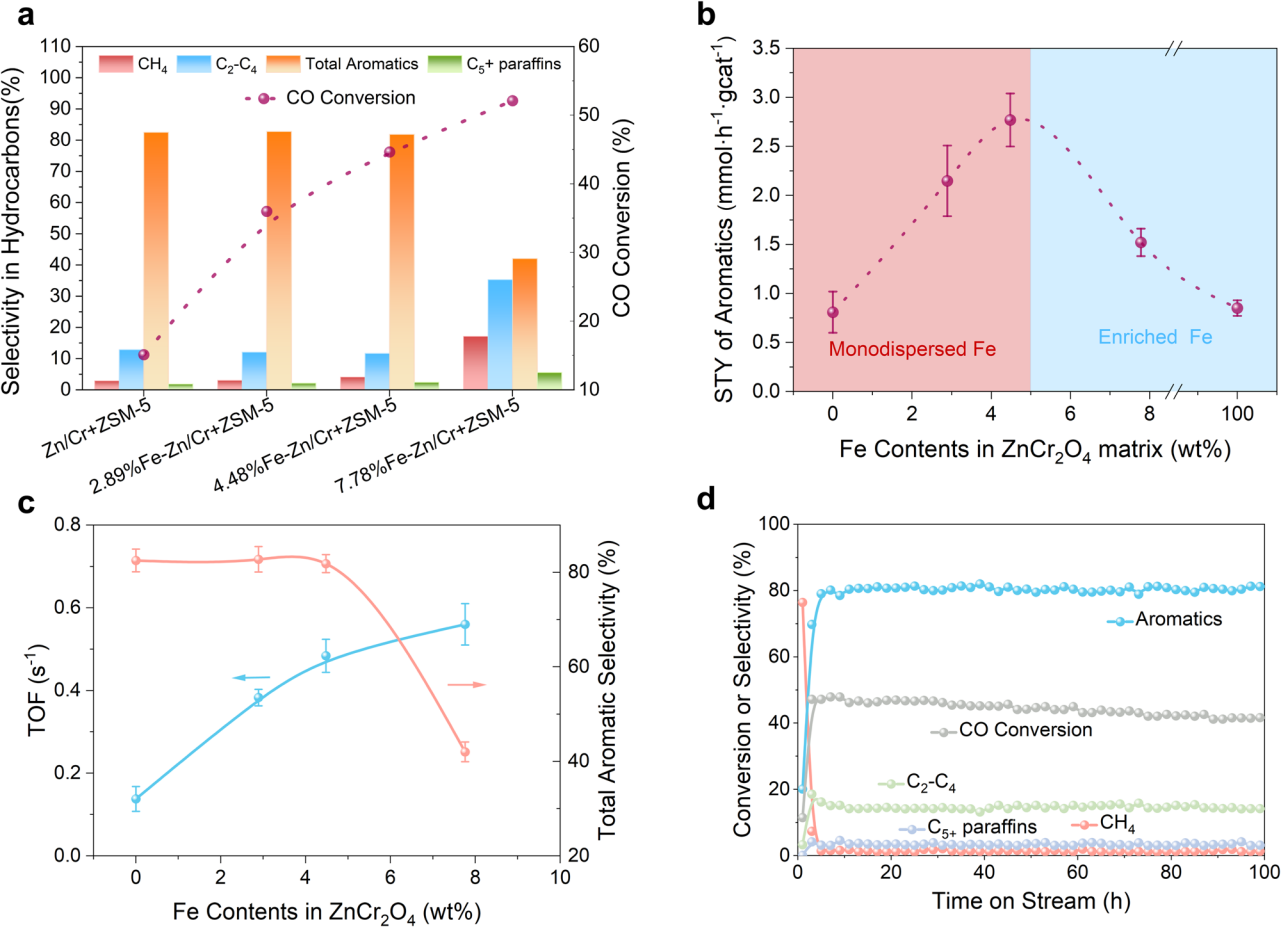
Catalyst evaluation of monodispersed and enriched Fe. **a** Catalyst evaluation of pristine ZnCr_2_O_4_ and different Fe-Zn/Cr with H-ZSM-5; reaction conditions: 350 °C, 2.0 MPa, space velocity = 600 ml h^−1^·g_cat_^−1^, CO:H_2_ = 1:1. **b** The space-time yield (STY) of aromatics for different catalysts. **c** TOF and aromatic selectivity as functions of Fe contents in ZnCr_2_O_4_ and reaction conditions are similar to A. **d** Stability test of 4.48% Fe-Zn/Cr + H-ZSM-5 catalytic system for 100 h. All the data in (**b**, **c**) were collected three times, and the error bars represent the standard deviation.

To validate the above prospect on the superior stability of monodispersed Fe in ZnCr_2_O_4_ through theoretical prediction, different Fe-Zn/Cr oxides were coupled with H-ZSM-5 as tandem catalysts for selective syngas conversion to aromatics. Figure 2a shows the catalyst evaluation of Fe-Zn/Cr vs. pristine ZnCr_2_O_4_ (denoted as Zn/Cr) at reaction conditions of 2.0 MPa (CO:H_2_ = 1:1), 350 °C, and 600 ml h^−1^ g_cat_^−1^. CO conversion increases from 13% using pristine Zn/Cr to 44% using 4.48% Fe-Zn/Cr with monodispersed Fe sites; meanwhile, the total aromatic selectivity remains as high as 80% with various alkanes as minor side products. Since the conversion processes in tandem, i.e., syngas to oxygenates and oxygenates to aromatics, are spatially separated, the enhanced CO conversion is attributed to the activation effect of Fe dopants on CO and H_2_ to accelerate oxygenate formation. In situ diffuse reflectance infrared Fourier transform spectroscopy results shows that signal intensities of oxygenate intermediates are at least an order of magnitude higher on 4.48% Fe-Zn/Cr than on pristine Zn/Cr, corroborating the above analysis (Supplementary Fig. 13). 7.78% Fe-Zn/Cr with enriched Fe increases the CO conversion to 52% but sacrifices the total aromatic selectivity from 80 to 42%, along with significantly higher yields of CH_4_ and C_2_–C_4_ alkanes. Since these alkanes are typical products of Fe_*x*_C_*y*_-catalyzed syngas conversion^24,25^, we characterized spent 7.78% Fe-Zn/Cr using HADDF-STEM and observed Fe_*x*_C_*y*_ phases in accordance (Supplementary Fig. 14). The Fe_*x*_C_*y*_ phases likely originated from the Fe–O–Fe structure present in the enriched Fe parts of 7.78% Fe-Zn/Cr and this is rationalized by the negative formation energy of carbides from ZnFe_2_O_4_ in former theoretical prediction.

As a typical tandem reaction over a bifunctional OX-ZEO catalyst, it has been reported that low temperature and high pressure are crucial to aromatics selectivity and CO conversion, respectively^26,27^. In this study, these two conditions were also tested with monodispersed Fe on ZnCr_2_O_4_. A low reaction temperature of 275 °C was previously found to increase the single-pass aromatic selectivity to ~82–84% using unmodified ZnCr_2_O_4_ + H-ZSM-5 catalysts; while 4.48% Fe-Zn/Cr profoundly promotes the aromatic selectivity up to 88–91%, a value that has rarely been achieved (Supplementary Fig. 15a, b). It means that even at a low temperature, the monodispersed Fe is able to activate CO and H_2_ to produce oxygenate intermediates, which further diffuse into the pores of zeolites to initiate C–C coupling and subsequent aromatics formation^22,28^. In addition to low-temperature performance, ZnCr_2_O_4_ with monodispersed Fe also exhibits outstanding high-pressure performance (Supplementary Fig. 15c, d). The CO conversion increases remarkably from 44 to 68%, whereas selectivity towards CH_4_ and C_2_–C_4_ side products is only marginally increased by **<**1%. By analyzing the Fe contents and space-time yields (STYs) of aromatics, it is found that STYs increase with higher Fe amounts only when Fe remains monodispersed and once Fe is further enriched to form Fe–O–Fe structure prone to carbonization, STYs of aromatics deteriorate due to the emergence of conventional Fischer–Tropsch pathways (Fig. 2b and Supplementary Fig. 16)^29^. This trend is also notable when projecting TOF and total aromatic selectivity to the Fe content; monodispersed Fe increases the TOF of syngas conversion and maintains high selectivity towards aromatics, whereas enriched Fe sacrifices the aromatic selectivity despite the higher TOF (Fig. 2c). As shown in Supplementary Table 4, the outstanding ability of our monodispersed Fe-Zn/Cr based OX-ZEO catalysts to simultaneously achieve high CO conversion and high aromatics selectivity is further illustrated through comparison with several recently reported catalysts for syngas conversion process^30–34^. The long-term stability test of 4.48% Fe-Zn/Cr + H-ZSM-5 shows stable performance for more than 100 h at 350 °C and 2.0 MPa. As shown in Fig. 2d, the aromatics selectivity remains larger than 80 wt% and CH_4_ within 2 wt%, and CO conversion decreases from 47 to 41%.

### Mechanistic investigation of monodispersed Fe in enhanced catalytic activity

**Fig. 3:**
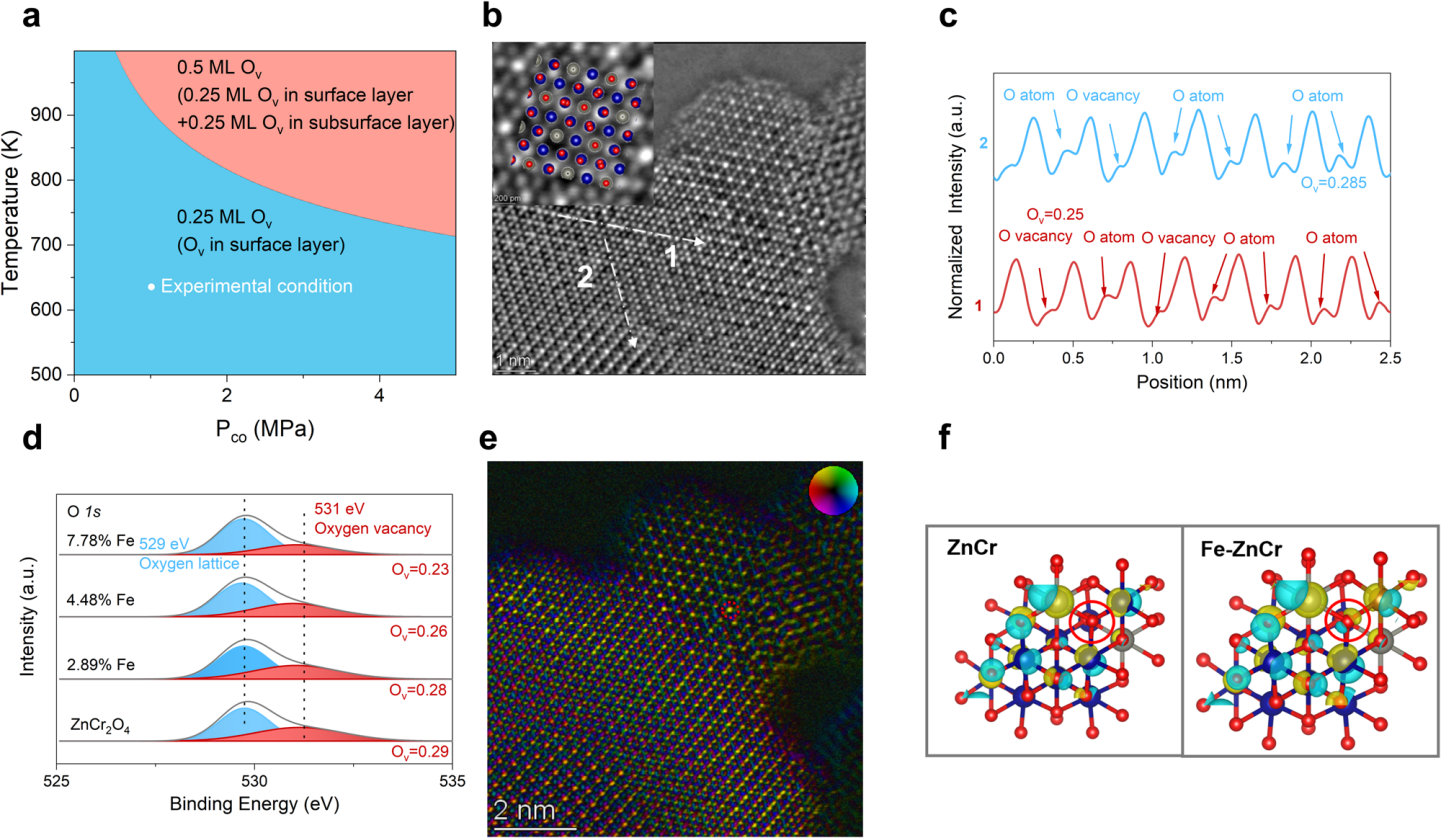
Understanding the role of monodispersed Fe in catalytic activity. **a** Thermodynamic phase diagram of Fe-doped ZnCr_2_O_4_ (111) surfaces at varying temperatures and CO partial pressures (*P*_CO_) **b** iDPC-STEM images of 4.48% Fe-Zn/Cr. **c** iDPC-STEM intensity profiles along the corresponding dashed lines in B. **d** O *1s XPS* spectra of ZnCr_2_O_4_ and different Fe-Zn/Cr samples. **e** Projected electric field strength and vector map of 4.48% Fe-Zn/Cr. **f** Differential charge density (cyan and yellow represent charge depletion and accumulation, respectively; the cutoff of the density-difference isosurface is 0.11e Å^−3^).

The above catalysis evaluation demonstrates the unique ability of monodispersed Fe to catalyze syngas to form aromatics when coupled with H-ZSM-5. To understand the origin of such enhanced activity, we further performed a series of combinary mechanistic investigations. Previous demonstrations have suggested surface O_V_ as possible active sites for CO activation^35,36^. However, there is a lack of high-precision characterization of O_V_^37^. DFT calculations were first performed to assess the equilibrium O_V_ concentration in monodispersed Fe-Zn/Cr samples. Using spinel (111) surface as a model surface, the formation energies of O_V_ with different concentrations and spatial locations (i.e., surface and subsurface layer) were calculated through either CO or H_2_ reduction (calculation details are presented in Supplementary Information). Similar to a previous study, CO reduction was found to be the major driving force for O_V_ formation^13^ (Supplementary Fig. 17). The phase diagram shows that O_V_ preferably presents in the surface layer with a concentration of 0.25 monolayer (ML) under the experimental condition (Fig. 3a). The atomic evidence of surface O_V_ was obtained by using the integrated differential phase-contrast STEM (iDPC-STEM) technique. Figure 3b depicts the (111) surface of 4.48% Fe-Zn/Cr and some O_V_ can be identified with atomic precision. A micro quantitative intensity statistical analysis was subsequently performed by applying a line scan to the iDPC-STEM images. The non-periodic variations in image contrast relating to O demonstrate the existence of O_V_ and its concentration is estimated to be 25–28%, similar to the theoretically predicted O_V_ concentration (Fig. 3c). O 1s XPS spectra of different Fe-Zn/Cr samples further show that Fe doping only alters surface O_V_ concentration by insignificant amounts (Fig. 3d). Electron paramagnetic resonance spectroscopy also quantitatively evidences the existence of O_V_ (Supplementary Fig. 18a, b). It is found that there are not much changes in the O_V_ content.

Since the O_V_ concentration remains marginally affected by Fe doping, it might not be the reason for the enhanced catalytic activity. We then focused on the effect of local electronic structures and performed a charge density analysis by combining electric field imaging and theoretical calculations. As shown in Fig. 3e, some local bright spots (in red circle) appear despite the surrounding uniform intensity, implying the possible charge transfer between isolated Fe and surrounding O and O_V_^38^. This is corroborated by the theoretically calculated charge density (Fig. 3f). Clearly, charges on O_V_ and O nearest to the Fe dopant increase in Fe-doped ZnCr_2_O_4_ with respect to pristine ZnCr_2_O_4_. The locally modulated charge density could account for the difference in catalytic activity between monodispersed Fe-Zn/Cr and Zn/Cr. A series of experiments were conducted to prove the promoted activation effect of Fe dopants on CO and H_2_ (Supplementary Figs. 19, 20).

To further demonstrate the solo metal oxide performance, we carried out the catalyst evaluation over the metal oxides at the conditions of 350 °C, 600 ml hr^−1^ g_cat_^−1^, and 2.0 MPa. As shown in Supplementary Fig. 21a, the CO conversion increases from 4.7 to 38.4% as the catalyst changes from pristine ZnCr to 7.78% Fe-ZnCr. Interestingly, the main product changes from CH_3_OH to CH_4_ when introducing Fe on the surface of ZnCr_2_O_4_. However, at the temperature of 350 °C, it is unlikely to form the aromatics with CH_4_ based on either experiment using H-ZSM-5 catalysts or thermodynamic equilibrium analysis (Supplementary Fig. 21b)^39,40^. Given the fact that Fe-ZnCr catalysts did show a product selectivity towards aromatics when mixed with ZSM-5, it is deduced that ZSM-5 in the composite catalyst can utilize certain reaction intermediates prior to CH_4_ formation along the CO hydrogenation pathways on the oxide part. The generally higher CO conversion observed for Fe-ZnCr/ZSM-5 composite catalyst than the solo Fe-ZnCr catalyst further confirms that the aromatization of reaction of these intermediates in the pores of ZSM-5 should have a higher rate than the further hydrogenation of these intermediates towards CH_4_ or CH_3_OH on the oxide surface.

**Fig. 4:**
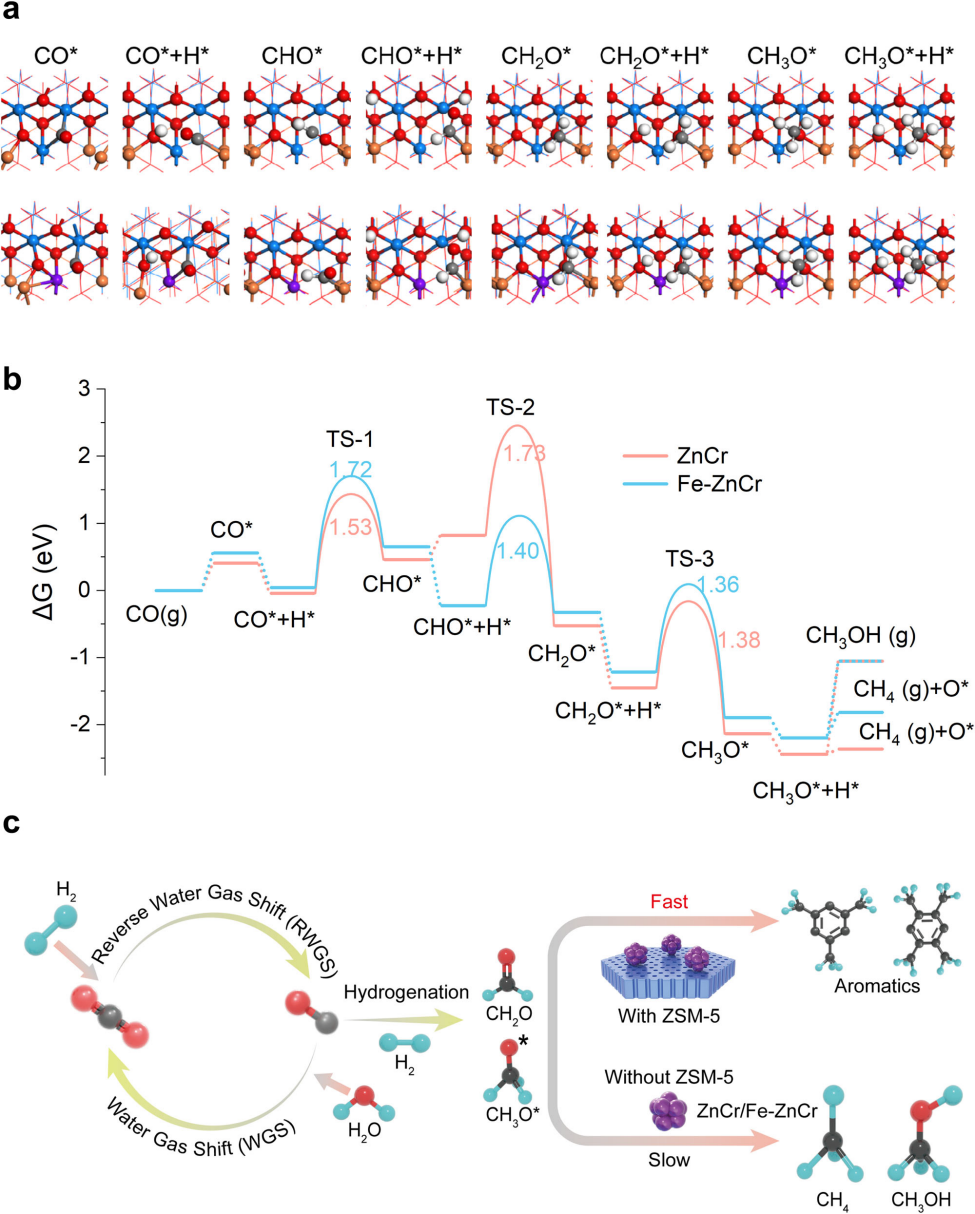
The mechanism study of monodispersed Fe and pristine ZnCr_2_O_4_ in syngas conversion. **a** The atomic structures of the syngas conversion to CH_3_OH/CH_4_. The reaction images are shown in the inset of **a**; Zn: orange, Cr: blue, Fe: purple, O: red, C: gray, and H: white. **b** Gibbs free energy diagrams of syngas conversion to CH_3_OH/CH_4_ on pristine and Fe-doped ZnCr_2_O_4_ (111) surfaces with 1/4 ML O_V_ at 623 K, 2.0 MPa. **c** Mechanistic diagram of syngas conversion to aromatics over composite catalysts.

CO hydrogenation reaction pathways were investigated using DFT calculations. By taking the aforementioned structural characterization and theoretical analysis into consideration, undoped and isolated Fe-doped ZnCr_2_O_4_ (111) surfaces with 1/4 ML O_V_ on the surface were constructed for simulations. The gas phase energies of CO and H_2_ are adjusted using the actual experimental conditions. On both surfaces, our calculations support the stepwise hydrogenation mechanism in syngas conversion^13,15,41^ (Fig. 4a). In more detail, the reaction starts with CO adsorption at O_V_ (Fig. 4b). Next, H_2_ dissociates near the surface O_V_ and H* is adsorbed on top of an O near Fe (16d). Subsequently, adsorbed CO* is stepwise hydrogenated to form formyl (CHO*), formaldehyde (CH_2_O*), methoxy (CH_3_O*), and finally CH_3_OH/CH_4_. It is found that pristine ZnCr_2_O_4_ (111) with O_V_ is mainly limited by the weak adsorption of H* with the presence of CHO*, as well as the strong adsorption of CH_3_O*. The incorporation of isolated Fe effectively stabilizes the state of CHO* + H*, making the hydrogenation step to form H_2_CO* energetically exothermic, while destabilizes the state of CH_2_O* + H*, thereby facilitating the formation of CH_3_O*. Besides, according to Fig. 4b, c, the formation of CH_3_OH and CH_4_ on the oxide catalysts bifurcates from CH_3_O*, both of which are endothermic with reaction energies as high as 1.14 and 0.38 eV, respectively. These processes are even less thermodynamically feasible than the prior endothermic steps to form H_2_CO* from CO. Previous theoretical studies also suggest that the last step of hydrogenation CH_3_O* to CH_3_OH or CH_4_ has the highest kinetic barrier^13^. Such barriers may result in the low CO conversion on solo oxide catalysts with the absence of ZSM-5 as the aromatization pathway is eliminated and the oxygenate intermediates can only be reduced to CH_3_OH or CH_4_. Thus, in a tandem system of Fe-ZnCr+ZSM-5, the reaction pathway of forming CH_4_ on solo Fe-ZnCr is circumvented through the alternative aromatization pathway that consumes oxygenate intermediates. From both aspects, monodispersed Fe improves the ability of pristine ZnCr_2_O_4_ to produce key oxygenate intermediates of H_2_CO and CH_3_O* from syngas. These key intermediates further participate in complicated oxygenate-to-aromatics reactions occurring in the pores of H-ZSM-5^42^.

In summary, we demonstrated that the spinel structure of ZnCr_2_O_4_ can spontaneously disperse strong self-interaction metal, Fe, due to the thermodynamically favorable interaction between Fe and ZnCr_2_O_4_, showing remarkable performance in syngas-to-aromatic reaction. By combining high-precision microscopic and macroscopic characterizations, as well as theory calculations, monodispersed Fe was found to activate the surrounding O_V_, thereby facilitating the CO and H_2_ activation while the formation of Fe_x_C_y_ under a reductive atmosphere was avoided. The TOF increased from 0.14 to 0.48 s^−1^ without sacrificing the aromatic selectivity by leveraging monodispersed Fe. The monodispersed Fe/ZnCr_2_O_4_ catalyst showed a 81.4% aromatic selectivity at a single-pass CO conversion of 68.4% by adopting an optimized reaction condition, outperforming other Fe-based catalysts for the syngas-to-aromatic reaction. DFT calculations revealed that the monodispersion of Fe in 16d sites lowered the energy barrier of a key step of formyl hydrogenation from 1.73 to 1.40 eV, thereby accelerating the formation of C_1_ oxygenate intermediates, i.e. precursors for aromatic formation in the pores of H-ZSM-5. This study provides a rationally tailored prototype catalyst with highly stable monodispersed active metal species for syngas chemistry, demonstrating targeted catalytic properties and inhibition of metal carbide formation.

## Methods

### Synthesis of doping Fe in ZnCr_2_O_4_ catalyst and pristine ZnCr_2_O_4_

ZnCr_2_O_4_ oxide was prepared by the co-precipitation method. Appropriate amounts of zinc nitrate Zn(NO_3_)_2_.6H_2_O and chromium nitrate Cr(NO_3_)_3_.9H_2_O (Zn:Cr = 0.5) were dissolved in distilled water to prepare a metal salt solution. An aqueous solution of ammonia (6 M) and ammonium bicarbonate NH_4_HCO_3_ (0.075 M) was used as the precipitating agent. Precipitates of metal oxide precursors were realized by adding metal salt solution and precipitating agent together in a three-neck round bottom flask while keeping the solution temperature at 70 °C and pH at 7. The aging process was done for 24 h at 90 °C. Both precipitation and aging processes were done in an N_2_ environment (100 ml/min). The obtained precipitates were then collected through filtration (washed several times with deionized (DI) water to remove the mother liquor) and dried overnight in the oven at 110 °C. Calcination was performed at 450 °C for 2 h to transform the precipitates into metal oxides.

The doping of Fe in ZnCr_2_O_4_ catalyst was achieved by employing an impregnation method. First, 2.3 g ZnCr_2_O_4_ was dropped into the solution made by dissolving 0.25/0.417/1.0 g ferric citrate (FeC_6_H_5_O_7_) in the 5 ml of DI water, then the solution was kept stirring at the temperature of 60 °C for 2 h. The mixture, after being stirred, was dried at 110 °C overnight and followed by calcination at the temperature of 300 °C for 2 h. Different weights of FeC_6_H_5_O_7_ correspond to Fe-Zn/Cr samples with different Fe doping amounts.

### Synthesis of H-ZSM-5 catalyst

Nano-sized H-ZSM-5 was prepared by using the hydrothermal method. Appropriate amounts of Tetraethyl orthosilicate (TEOS (11.2 g)), Tetrapropylammonium hydroxide (TPAOH (13.1 g)), Aluminum nitrate (Al(NO_3_)_3_·9H_2_O (0.3 g)), Sodium hydroxide (NaOH (0.1 g)), Propan-2-ol (IPA (0.1 g)), and urea (2 g) were dissolved in DI water (18.4 g) under vigorous stirring for at least 6 h, at room temperature. The solution was transferred into a Teflon-lined stainless steel autoclave reactor that was further placed in a temperature-programmed oven. For crystallization, the temperature of the oven was raised from room temperature to 180 °C with a heating rate of 15 °C/h and held at 180 °C for 48 h. Once the crystallization is complete, the temperature of the autoclave reactor was quenched rapidly using a cold water bath. The obtained crystals were then collected through the filtration process (washed several times with DI water to remove the mother liquor) and dried overnight in the oven at 90 °C. To remove the templating agent TPAOH, the crystals were calcined in air at 550 °C for 5 h. Using a cation exchange method (step repeated at least three times), the Na-form of ZSM-5 (i.e., 10 g) was converted to NH_4_-ZSM-5 after being dispersed in a 1 M NH_4_NO_3_ solution (i.e., 100 mL) under vigorous stirring for 6 h. Finally, NH_4_-ZSM-5 was transformed into H-ZSM-5 through a simple calcination step performed at 550 °C for 5 h in the environment of air. The resulting powder was denoted as H-ZSM-5.

### Catalytic tests

The syngas conversion was performed on a high-pressure fixed-bed flow reactor designed by Xintong Si company, Beijing. Typically, the catalyst (1.0 g) with grain sizes of 250–600 μm (30–60 mesh) was loaded in a titanium reactor (inner diameter, 10 mm). The syngas with an H_2_/CO ratio of 1:1 was employed in this work and Ar with a concentration of 4% contained in the syngas was used as an internal standard for the calculation of CO conversion. The flow rate of syngas was controlled to be as constant as 10 cm^3^/min (293 K, 101 kPa) for each reaction. The syngas pressure and the reaction temperature were typically 2.0—6.0 MPa and 623 to 700 K, respectively. The product selectivity was calculated on a molar carbon basis for CO hydrogenation.

CO conversion (Conv_CO_) was calculated on a carbon atom basis, i.e.,

1 $${{{{{{{\mathrm{Conv}}}}}}}}_{{{{{{{\mathrm{CO}}}}}}}}=\frac{{{{{{{{\mathrm{CO}}}}}}}}_{{{{{{{\mathrm{inlet}}}}}}}}-{{{{{{{\mathrm{CO}}}}}}}}_{{{{{{{\mathrm{outlet}}}}}}}}}{{{{{{{{\mathrm{CO}}}}}}}}_{{{{{{{\mathrm{inlet}}}}}}}}}\times 100\%$$

where CO_inlet_ and CO_outlet_ in Eq. (1) represented moles of CO at the inlet and outlet, respectively

The turn of frequency is based on the equation

2 $${{{{{{\mathrm{TOF}}}}}}},\;{s}^{-1}=\frac{{n}_{{{{{{{\mathrm{syngas}}}}}}}}^{{{{{{{\mathrm{inlet}}}}}}}}-{n}_{{{{{{{\mathrm{syngas}}}}}}}}^{{{{{{{\mathrm{outlet}}}}}}}}}{{n}_{{{{{{{\mathrm{active}}}}}}}\;{{{{{{\mathrm{sites}}}}}}}}}$$

The selectivity of individual hydrocarbon C_n_H_m_ (Sel-C_n_H_m_) among hydrocarbons (free of CO_2_) in Eq. (3) was calculated according to

3 $${{{{{{{\mathrm{Sel}}}}}}}}_{{CnHm}}=\frac{{n}_{{cnHm}\;{{{{{{\mathrm{outlet}}}}}}}}}{\mathop{\sum }\limits_{1}^{n}{n}_{{CnHm}\;{{{{{{\mathrm{outlet}}}}}}}}}\times 100\%$$

Little C_12+_ hydrocarbons were detected. The selectivity to oxygenates was below 1%C and therefore neglected.

CO2 selectivity (Sel_co2_) was calculated according to

4 $${{{{{{{\mathrm{Sel}}}}}}}}_{{{{{{{\mathrm{CO}}}}}}}2}=\frac{{{{{{{{\mathrm{CO}}}}}}}2}_{{{{{{{\mathrm{outlet}}}}}}}}}{{{CO}}_{{{{{{{\mathrm{inlet}}}}}}}}-{{CO}}_{{{{{{{\mathrm{inlet}}}}}}}}}$$

where CO_inlet_ and CO_outlet_ in Eq. (4) represented moles of CO at the inlet and outlet, respectively, while CO_2outlet_ in Eq. (4) denotes moles of CO_2_ at the outlet.

The carbon balance was calculated according to the following formula:

5 $${C}_{{{{{{{\mathrm{balance}}}}}}}}=\frac{{{{{{{{\mathrm{CO}}}}}}}2}_{{{{{{{\mathrm{outlet}}}}}}}}+{n}_{{CnHm}}}{{{{{{{{\mathrm{CO}}}}}}}}_{{{{{{{\mathrm{inlet}}}}}}}}-{{{{{{{\mathrm{CO}}}}}}}}_{{{{{{{\mathrm{outlet}}}}}}}}}\times 100\%$$

The carbon balance over the composite catalysts was between 97 and 100%.

One gram of catalyst (a physical mixture of an equal mass of ZnCr or Fe/ZnCr and H-ZSM-5) supported on top of silica sand and quartz wool was placed within the isothermal region of the reactor. Before catalytic evaluation, the catalyst was reduced in situ in an H_2_ environment at 350 °C for 2 h.

### Characterizations

#### HADDF-STEM, SEM, TEM

The morphologies of samples were performed by field-emission scanning electron microscopy (FESEM, SU-8010) and transmission electron microscopy (TEM, HT7700 120 kV). The atomic scale STEM (HAADF) experiments were performed using a Cs-corrected scanning transmission electron microscope (FEI Titan Cubed Themis G2 300) operated at 300 kV with a convergence semi-angle of 23.6 mrad and the beam current was lower than 30 pA. The microscope was equipped with a DCOR+ spherical aberration corrector for the electron probe, which was aligned before the experiments using a standard gold sample. The dwell time of probe scanning was 32 μs for all the EDS mapping image. The dwell time was 1 µs per pixel with a map size of 256 × 256 pixels; a complete process of EDS mapping took roughly 0.5 h to reach an appropriately high signal-to-noise ratio. The following aberration coefficients were measured as: A1 = 694 pm; A2 = 22.1 nm; B2 = 6.12 nm; C3 = 82.5 nm; A3 = 63.6 nm; S3 = 136 nm; A4 = 2.13 μm, D4 = 4.54 μm, B4 = 1.71 μm, C5 = −49.2 μm, A5 = 222 μm, S5 = 102 μm, and R5 = 17.3 μm. Atomic-resolution iDPC-STEM was also operated at 300 kV with a convergence semi-angle was 15 mrad. The beam current was set between 1 and 0.5 pA. The crystal structures of samples were determined by X-ray diffraction (Bruker D8). The iDPC-STEM experiments were performed using a Cs-corrected scanning transmission electron microscope (FEI Titan Cubed Themis G2 300) operated at 300 kV. The microscope was equipped with a DCOR+ spherical aberration corrector for the electron probe, which was aligned before the experiments using a standard gold sample. The following aberration coefficients were measured as: A1 = 1.41 nm; A2 = 11.5 nm; B2 = 22.2 nm; C3 = 2.05 μm; A3 = 525 nm; S3 = 177 nm; A4 = 8.81 μm, D4 = 2.39 μm, B4 = 13.2 μm, C5 = −3.95 mm, A5 = 295 μm, S5 = 111 μm, and R5 = 102 μm. The convergence semi-angle was 15 mrad, the beam current was lower than 0.5 pA (the measurement was limited by the precision of the Faraday cup), the collection angle was 4–22 mrad, and the dwell time of probe scanning was 32 μs. X-ray photoelectron spectroscopy (XPS) of samples was tested on Escalab 250 Xi XPS with an Al Kα X-ray resource (Thermo Fisher Scientific). The Brunauer-Emmett-Teller surface area of samples were performed by a specific surface area and mesoporous analyzer (TriStar II).

#### XRD

X-ray diffraction (XRD) was measured on a PANaly-tical Empyrean-100 equipped with a Cu Kα radiation source (λ = 1.5418 Å), operated at 40 mA and 40 kV. XRD patterns were recorded in the range of 2 theta = 10–90°. The crystal size was estimated using the Scherrer equation.

#### ICP-OES

The elemental content was measured using an inductively coupled plasma optical emission spectrometer (ICP-OES).

#### EPR

paramagnetic resonance (EPR) spectra were collected at 7 K on a Bruker A200 EPR spectrometer operated at the X-band frequency using power 1.0 mW, modulation amplitude 4.00 G, and receiver gain 10,000. The photoluminescence (PL) spectra were measured using QM400 with a Xe-lamp as the excitation source at room temperature. The excitation wavelength was fixed at 290 nm.

#### H_2_-TPD

The H_2_-temperature-programmed desorption profiles (H_2_-TPD) were performed on a Micromeritics AutoChem II 2920 analyzer equipped with mass spectrometry, argon, and 10% H_2_–Ar were used for reference and reduction, respectively.

#### H_2_-D_2_ exchange

The H_2_-D_2_ exchange experiment was performed with a temperature-programed reactor equipped with a mass spectrometer Omnistar. About 30 mg metal oxide catalyst was put into a quartz reaction tube. Then the reactor was heated at 5 °C/min to 400 °C in Ar atmosphere (20 ml/min) for pretreatment. The reaction gas (H_2_/D_2_/Ne = 2/2/1, 5 ml/min) was introduced into the system after cooling to room temperature. Finally, the tube was heated to 450 °C at 5 °C/min until 450 °C.

#### In situ drifts

The in situ diffuse reflectance infrared Fourier transform spectroscopy (DRIFTS) measurements were performed on a Bruker Tensor 27 instrument equipped with an MCT detector to detect the change of intensity of surface intermediate species. Typically, a diffuse reflectance infrared cell with a ZnSe window was loaded with 50 mg of the sample, which was pretreated with 30 mL min^−1^ of H_2_/N_2_ flow (H_2_/N_2_ = 1/5) under 0.1 MPa at 350 °C and followed by sweeping with pure N_2_ at a flow rate of 30 mL min^−1^. After that, the temperature was kept to 390 °C and the background spectrum was recorded. Then 5 mL min^−1^ of syngas (H_2_/CO/Ar = 47.5/47.5/5) was introduced into the infrared cell under 0.1 MPa at 350 °C and the in situ DRIFT spectra were acquired at 16 scans with a resolution of 4 cm^−1^.

#### XAFS

XAFS measurements at Fe K-edge in transmission mode were performed at the BL14W1 in Shanghai Synchrotron Radiation Facility (SSRF). The electron beam energy was 3.5 GeV and the stored current was 230 mA (top-up). A 38-pole wiggler with a maximum magnetic field of 1.2 T inserted in the straight section of the storage ring was used. XAFS data were collected using a fixed-exit double-crystal Si (111) monochromator.

The spectra were calibrated, averaged, pre-edge background subtracted, and post-edge normalized using the Athena program in the IFEFFIT software package. The Fourier transformation of the k2-weighted EXAFS oscillations, k2·χ(k), from k space to R space were performed over a range of 3.0–12.0 Å^−1^(Fe data) to obtain radial distribution function. EXAFS data were fitted by the Artemis program in IFEFFIT.

#### DFT methods

The structural optimizations were performed with density functional theory, with a periodic plane-wave implementation using Vienna ab initio Simulation Package (VASP) code^43^ The exchange-correlation energy was modeled by using Perdew–Burke–Ernzerhof (PBE) functional^44^ within the generalized gradient approximation (GGA). The projector augmented wave (PAW) pseudo-potentials^45^ were used to describe ionic cores. An energy cutoff of 450 eV was adopted. Gaussian smearing of 0.1 eV was applied to the orbital occupation during the geometry optimization and for the energy computations. The *U* values were taken from refs. 13,46 and ref. 47, respectively. To further validate the *U* values selected, we have also compared the results with the ones in Materials Project^48^, where the *U* values were obtained by fitting to experimental binary formation enthalpies as described in ref. 49. For reaction 1/2Cr_2_O_3_ + 3/4O_2_ → CrO_3_ and 1/3Fe_3_O_4_ + 3/8O_2_ → 1/2Fe_2_O_3_, the differences between our calculations and the ones from the Materials Project were 0.09 and –0.02 eV. The *U* values chosen were therefore considered reasonable. To properly describe the localized 3*d* electrons correlation, the DFT + *U* method was applied through the rotationally invariant approach^50^ with *U* = 3.3 eV for Cr and *U* = 5.0 eV for Fe. Spin polarization was applied to all systems calculated.

The adsorption energies were evaluated using six-layer 2 × 2 supercells with the bottom four layers constrained, and [3 × 3 × 1] Monkhorst-Pack *k*-point grids were used^51^ with a convergence threshold of 10^–6^ eV for the iteration in the self-consistent field (SCF). All structures were optimized until force components were less than 0.02 eV/Å. The vibrational frequencies of free molecules and adsorbates were calculated by using the phonon modules in the VASP 5.3 code. A standard thermodynamic correction was applied to determine the free energy corrections, including the correction of the effect from zero-point energy, pressure, inner energy, and entropy. The energy barrier of the transition state is converged by using PBE.

#### Energy definitions

The stability of Fe-doped ZnCrO_4_ and ZnFe_2_O_4_ with respect to Fe_5_C_2_ were determined with the following equations:

$$0.5{{{{{{\rm{ZnFe}}}}}}}_{2}{{{{{{\rm{O}}}}}}}_{4}+0.4{{{{{\rm{CO}}}}}}+1.9{{{{{{\rm{H}}}}}}}_{2}\to 0.2{{{{{{\rm{Fe}}}}}}}_{5}{{{{{{\rm{C}}}}}}}_{2}+0.5{{{{{\rm{ZnO}}}}}}+1.9{{{{{{\rm{H}}}}}}}_{2}{{{{{\rm{O}}}}}}$$

$${{{{{\rm{Fe}}}}}}/{{{{{{\rm{ZnCrO}}}}}}}_{{{{{{\rm{x}}}}}}}+0.4{{{{{\rm{CO}}}}}}+0.4{{{{{{\rm{H}}}}}}}_{2}\to {{{{{{\rm{ZnCrO}}}}}}}_{{{{{{\rm{x}}}}}}}+0.2{{{{{{\rm{Fe}}}}}}}_{5}{{{{{{\rm{C}}}}}}}_{2}+0.4{{{{{{\rm{H}}}}}}}_{2}{{{{{\rm{O}}}}}}$$

The Gibbs free energy change of the above reactions were adopted to quantify the likelihood of Fe-doped ZnCrO_4_ and ZnFe_2_O_4_ to form Fe_5_C_2_.

The substitution energy was computed using the following equations:

$${{{{{{\rm{Zn}}}}}}}_{{{{{{\rm{a}}}}}}}{{{{{{\rm{Cr}}}}}}}_{{{{{{\rm{b}}}}}}}{{{{{{\rm{O}}}}}}}_{{{{{{\rm{c}}}}}}}+0.5{{{{{{\rm{Fe}}}}}}}_{2}{{{{{{\rm{O}}}}}}}_{3}\to {{{{{{\rm{Zn}}}}}}}_{{{{{{\rm{a}}}}}}-1}{{{{{{\rm{FeCr}}}}}}}_{{{{{{\rm{b}}}}}}}{{{{{{\rm{O}}}}}}}_{{{{{{\rm{c}}}}}}}+{{{{{\rm{ZnO}}}}}}+0.25{{{{{{\rm{O}}}}}}}_{2}$$

$${{{{{{\rm{Zn}}}}}}}_{{{{{{\rm{a}}}}}}}{{{{{{\rm{Cr}}}}}}}_{{{{{{\rm{b}}}}}}}{{{{{{\rm{O}}}}}}}_{{{{{{\rm{c}}}}}}}+0.5{{{{{{\rm{Fe}}}}}}}_{2}{{{{{{\rm{O}}}}}}}_{3}\to {{{{{{\rm{Zn}}}}}}}_{{{{{{\rm{a}}}}}}}{{{{{{\rm{Cr}}}}}}}_{{{{{{\rm{b}}}}}}-1}{{{{{{\rm{FeO}}}}}}}_{{{{{{\rm{c}}}}}}}+0.5{{{{{{\rm{Cr}}}}}}}_{2}{{{{{{\rm{O}}}}}}}_{3}$$

Since the vibration entropy and the *pV* term contributions of solid phases are negligibly small, their Gibbs free energies can be approximated by their DFT total energy. The Gibbs free energy changes of the above equations were adopted to represent the corresponding substitution energy.

To determine the equilibrium O_v_ concentration in the ZnFeCrO system, the following equation was adopted to analyze the thermodynamic stability of the system:

$${{{{{{\rm{Zn}}}}}}}_{{{{{{\rm{a}}}}}}}{{{{{{\rm{FeCr}}}}}}}_{{{{{{\rm{b}}}}}}}{{{{{{\rm{O}}}}}}}_{{{{{{\rm{c}}}}}}}+{{{{{\rm{dCO}}}}}}/{{{{{{\rm{H}}}}}}}_{2}\to {{{{{{\rm{Zn}}}}}}}_{{{{{{\rm{a}}}}}}}{{{{{{\rm{FeCr}}}}}}}_{{{{{{\rm{b}}}}}}}{{{{{{\rm{O}}}}}}}_{{{{{{\rm{c}}}}}}-{{{{{\rm{d}}}}}}}+{{{{{{\rm{dCO}}}}}}}_{2}/{{{{{{\rm{H}}}}}}}_{2}{{{{{\rm{O}}}}}}$$

The Gibbs free energy changes of the above equation were computed at different pressures and temperatures for different O_v_ configurations and concentrations, and the one with the lowest energy change was considered the most stable phase under this condition. It has been found that the reductive ability of CO is stronger than H_2_ under the reaction condition presented, and therefore Fig. 3a in the main text shows the phase diagram under the CO condition.

## Supplementary information

Supplementary Information

## Data Availability

All data are available in the manuscript or the supplementary information. Additional data are available from the corresponding authors upon reasonable request.
