# Supplementary material for: Accelerating syngas-to-aromatic conversion via spontaneously monodispersed Fe in ZnCr2O4 spinel
Source: Nat Commun. 2022 Sep 22;13:5567. doi: 10.1038/s41467-022-33217-9 (PMC9500042; doi:10.1038/s41467-022-33217-9)
Supplement: Supplementary file 1 — Supplementary Information [file 41467_2022_33217_MOESM1_ESM.pdf]

Title: Accelerating Syngas-to-Aromatic Conversion via Spontaneously Monodispersed Fe in $ZnCr_2O_4$ Spinel

Authors: *Guo Tian*[1†], *Xinyan Liu*[2†], *Chenxi Zhang*[1*], *Xiaoyu Fan*[1], *Hao Xiong*[1], *Xiao Chen*[1*], *Zhengwen Li*[1], *Binhang Yan*[1], *Lan Zhang*[3], *Ning Wang*[3], *Hong-Jie Peng*[2*], *Fei Wei*[1*]

Affiliations:

1. Beijing Key Laboratory of Green Chemical Reaction Engineering and Technology, Department of Chemical Engineering, Tsinghua University, Beijing, China, 100084
2. Institute of Fundamental and Frontier Sciences, University of Electronic Science and Technology of China, Chengdu 611731, Sichuan, China
3. Faculty of Environment and Life, Beijing University of Technology, Beijing 100124, China

† These authors contributed equally to this work

* Corresponding authors Fei Wei, email: wf-dce@tsinghua.edu.cn, Chenxi Zhang, email: cxzhang@tsinghua.edu.cn, Xiao Chen, email: chenx123@tsinghua.edu.cn, Hong-Jie Peng: hjpeng@uestc.edu.cn.

XRD is considered as an efficient characterization to identify the spontaneously mono-dispersion metals on the surface of support. As shown in **Supplementary Figure 1 (a)**, all different wt% doping catalysts exhibited a similar phase condition with the $ZnCr_2O_4$ spinel structure and there are no significant changes between the surface index.

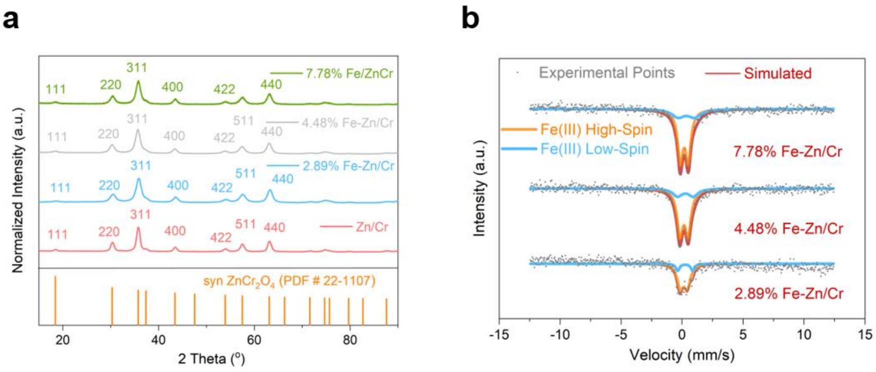

Supplementary Figure 1 | XRD and Mössbauer spectra characterizations of different Fe doping catalysts. (a) XRD pattern of different catalyst samples (b) Mössbauer spectra of mono-dispersion Fe and enrichment Fe samples

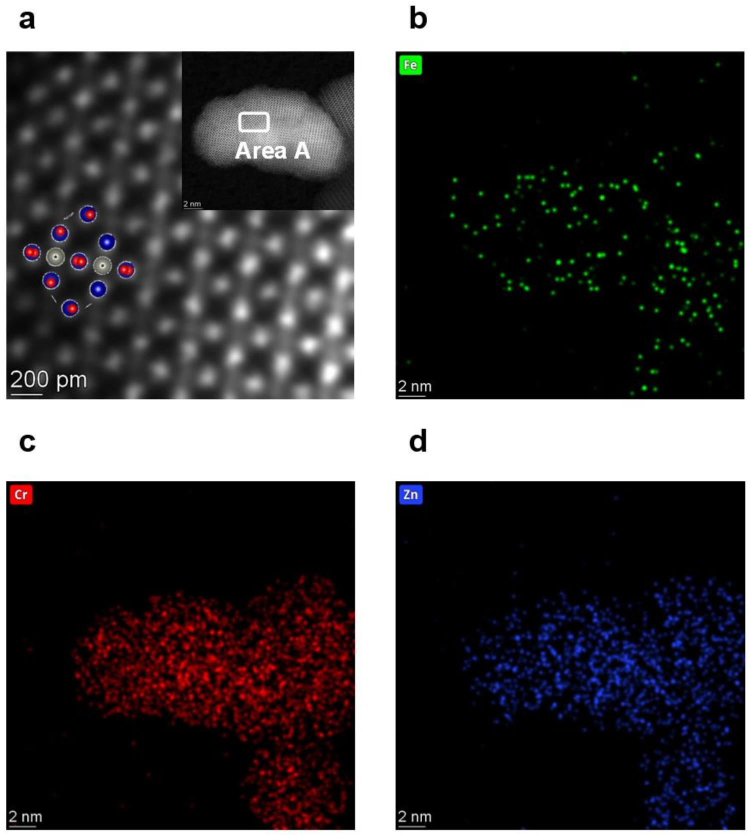

Supplementary Figure 2 | Structural characterizations of 2.89 wt% Fe-ZnCr. (a) HADFF-STEM image of [001] surface of the 2.89 wt% Fe in $ZnCr_2O_4$ matrix (b-d) EDS-mapping of the sample.

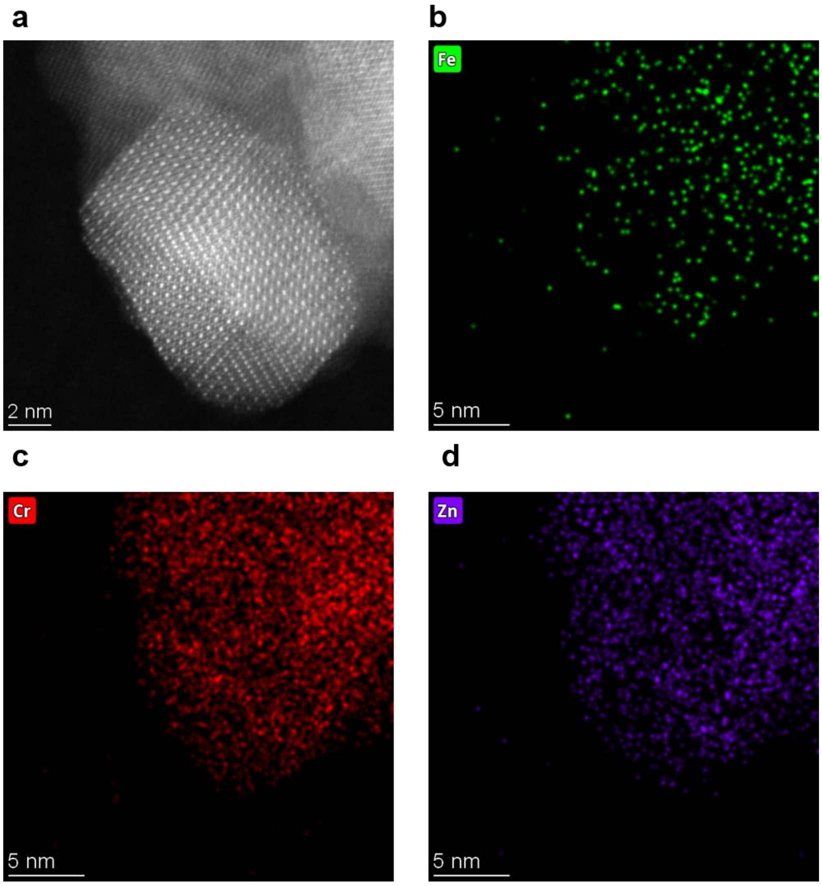

Supplementary Figure 3 | Structural characterizations of 4.48 wt% Fe-ZnCr. (a) HADFF-STEM image of [220] surface of the 4.48 wt% Fe in $ZnCr_2O_4$ matrix (b-d) EDS-mapping of the sample.

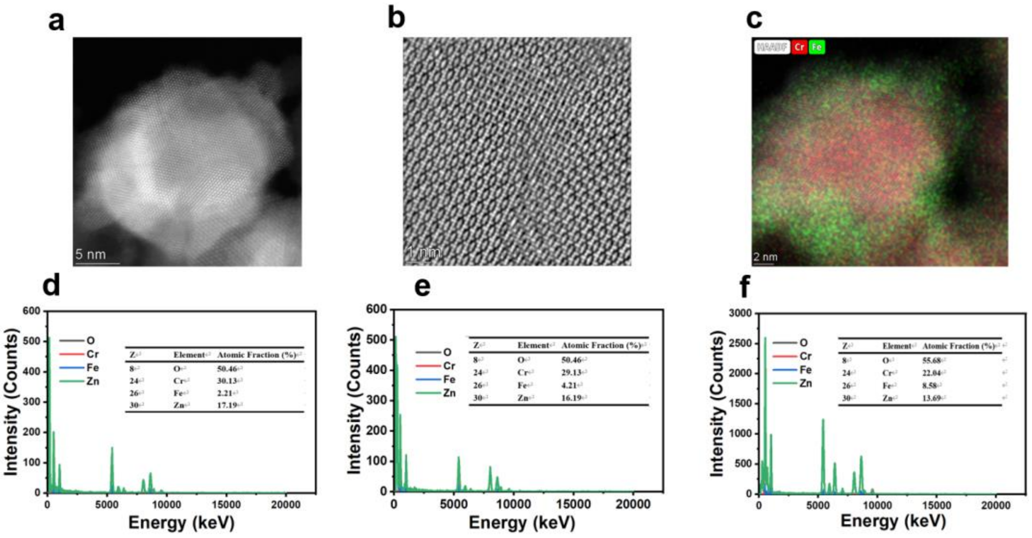

Supplementary Figure 4 | Structural characterizations of 7.78 wt% Fe-ZnCr. (a-b) HADDF-STEM images of 7.78 wt% Fe doping in $ZnCr_2O_4$ catalyst (c) HADDF-EDS-Mapping of 7.78 wt% Fe doping in $ZnCr_2O_4$ catalyst (d) Quantitative results of EDS-Mapping of 2.89 % wt Fe doping (e) Quantitative results of EDS-Mapping of 4.48% wt Fe doping (f) Quantitative results of EDS-Mapping of 7.78% wt Fe doping.

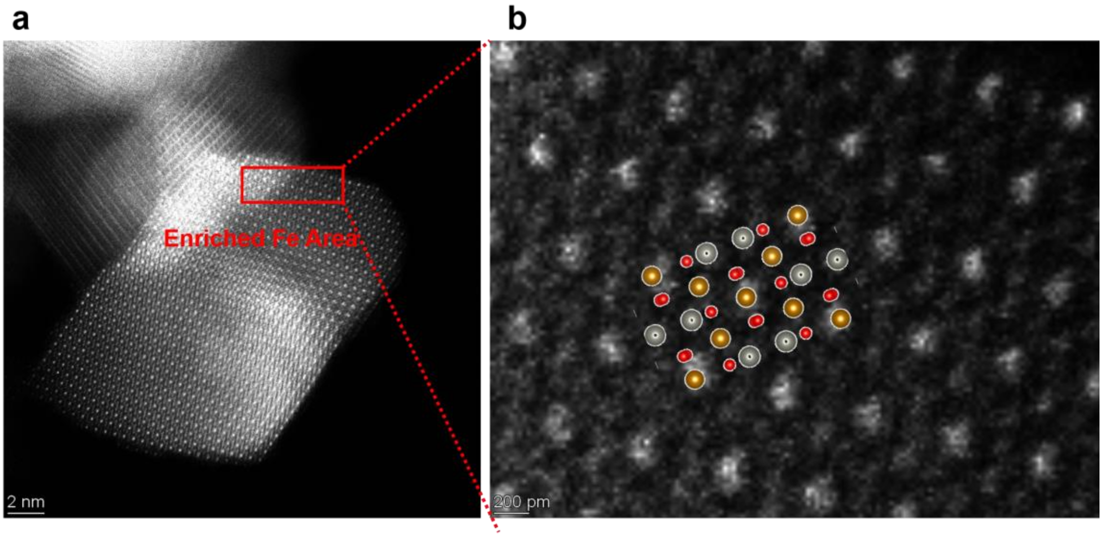

Supplementary Figure 5 | Structural characterization of enriched Fe area. (a) High resolution images of 7.78 wt% Fe-ZnCr. (b) High resolution images of enriched Fe area.

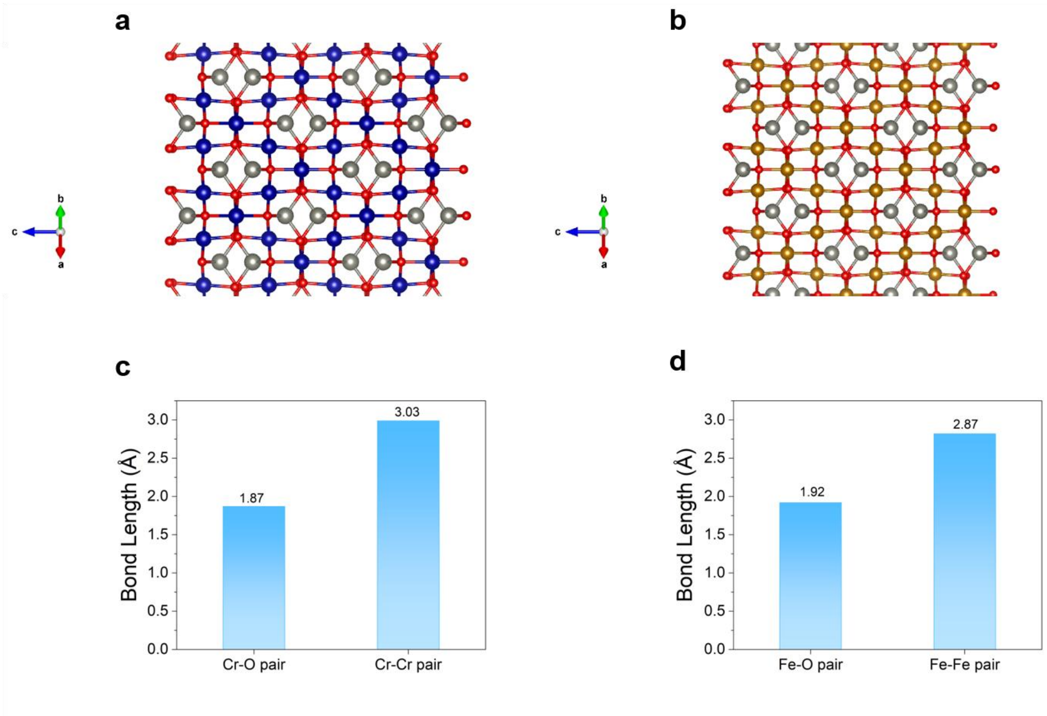

Supplementary Figure 6 | Surface information about [220] plane of $ZnCr_2O_4$ and $ZnFe_2O_4$. (a) [220] surface of the $ZnCr_2O_4$ spinel after optimization (b) [220] surface of the $ZnFe_2O_4$ spinel after optimization (c) Corresponding Cr-O and Cr-Cr pair bond lengths in $ZnCr_2O_4$ spinel (d) Corresponding Fe-O and Fe-Fe pair bond lengths in $ZnFe_2O_4$ spinel.

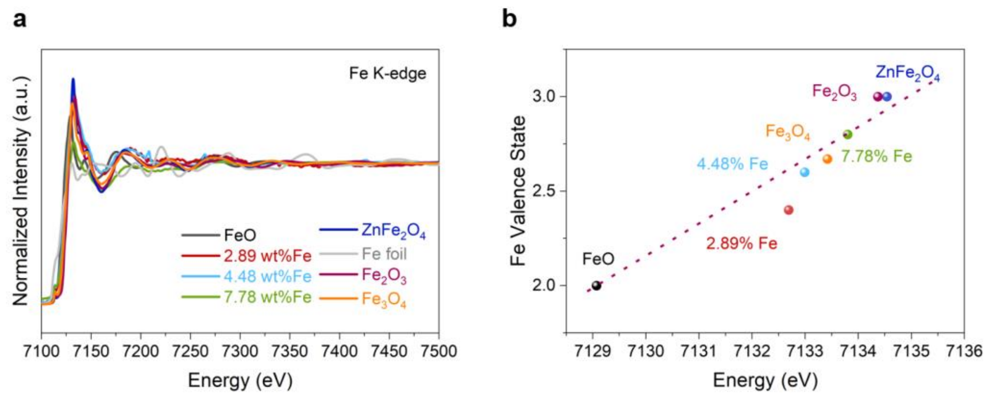

Supplementary Figure 7 | The mean chemical valences of Fe species. (a) Fe K-edge EXANES profiles of different Fe doping in $ZnCr_2O_4$ and some standard samples (b) The K-edge position (defined as the energy where the normalized absorption is 0.5) vs Fe oxidation states.

In **Supplementary Figure 8 (a)**, the major peak appears at 1.50 Å can be attributed to the Fe-O coordination and there are no significant divergences between the $ZnFe_2O_4$ and different Fe doping in $ZnCr_2O_4$ at the first peak. Remarkably, the second peak in the $ZnFe_2O_4$ and Fe doping samples show a little bit difference. **Supplementary Figure 8 (a)** shows the second peak for $ZnFe_2O_4$ is at 2.57 Å and 2.60 Å for 7.78 wt% Fe doping in $ZnCr_2O_4$, 2.71 Å for 2.89 wt% and 4.48 wt% Fe doping in $ZnCr_2O_4$. As shown in **Supplementary Figure 8 (b)**, the peak that appears at 711 eV in 7.78-wt% Fe-doped ZnCr2O4 is attributed to Fe–O–Fe, and the peak at 713 eV in those of 2.89 and 4.48 wt% is ascribed to Fe–O–Cr, which supports our deduction from FT-EXAFS

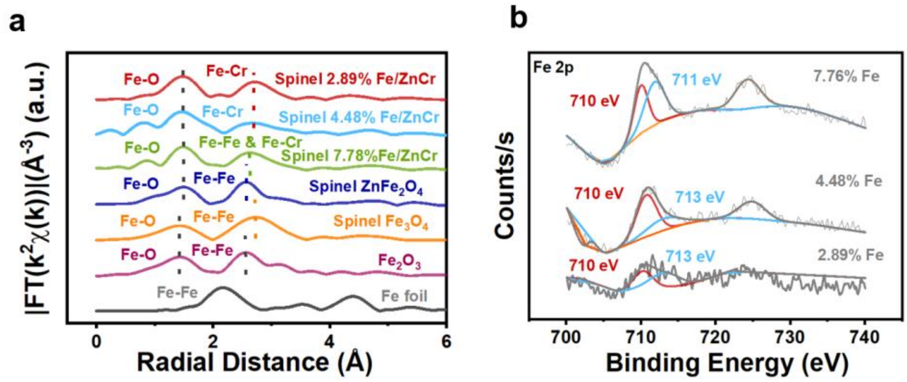

Supplementary Figure 8 | The local coordination information of Fe species. (a) Fe k^2-weighted FT-EXAFS profiles of different Fe doping in $ZnCr_2O_4$ and some standard samples (b) Fe 2p XPS spectra of different doping Fe in $ZnCr_2O_4$ samples.

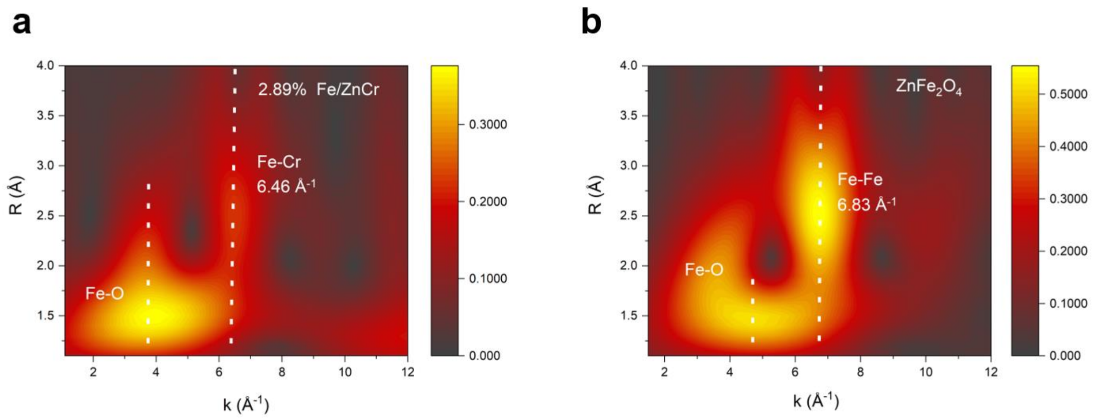

Supplementary Figure 9 | The WT-EXAFS spectra of Fe species. (a & b) Fe K-edge WT-EXAFS spectra of mono-dispersion Fe & $ZnFe_2O_4$ samples.

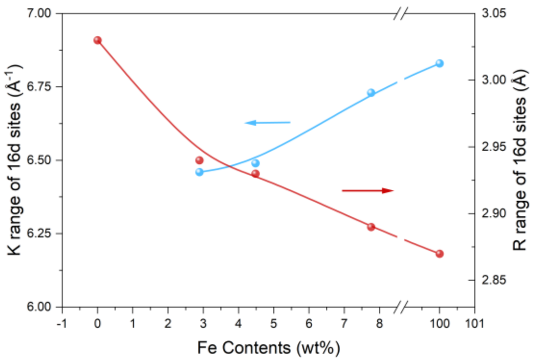

Supplementary Figure 10 | Bond length information over Fe contents. R range & K range over Fe contents profile.

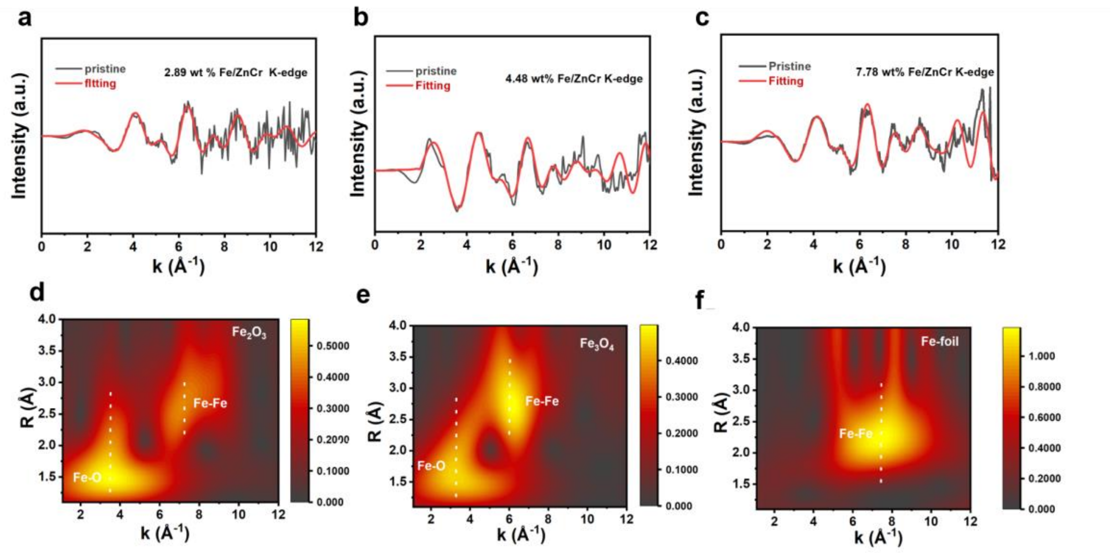

Supplementary Figure 11 | Simulated information of Fe species. (a) Pristine and fitting data of 2.89 wt% Fe doping in $ZnCr_2O_4$ (b) Pristine and fitting data of 4.48 wt% Fe doping in $ZnCr_2O_4$ (c) Pristine and fitting data of 7.78 wt% Fe doping in $ZnCr_2O_4$ (d) WT-EXAFS spectra of Fe_2O_3 samples (e) WT-EXAFS spectra of Fe_3O_4 samples (f) WT-EXAFS spectra of Fe-foil samples

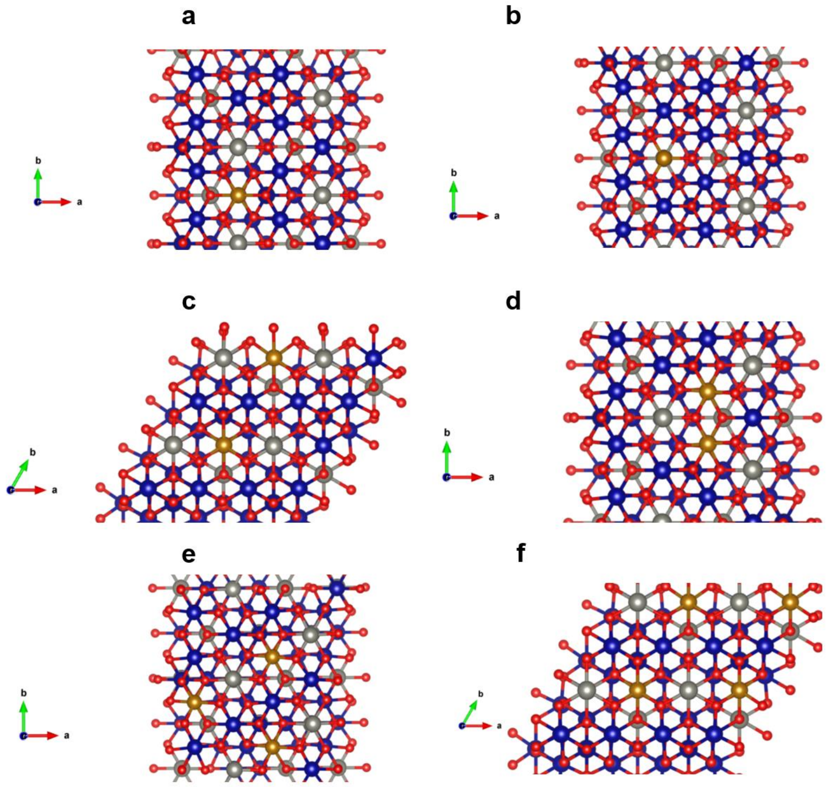

Supplementary Figure 12 | Atomic structure of Fe doping. (a) Structure model of Fe substitutes Cr sites(16d) (b) Structure model of Fe substitutes Zn sites (4a) (c) Structure model of Fe substitutes isolated 16d sites (d) Structure model of Fe substitutes adjacent 16d sites. (e) Structure model of triple Fe in 16d sites (f) Structure model of quadruple Fe in 16d sites.

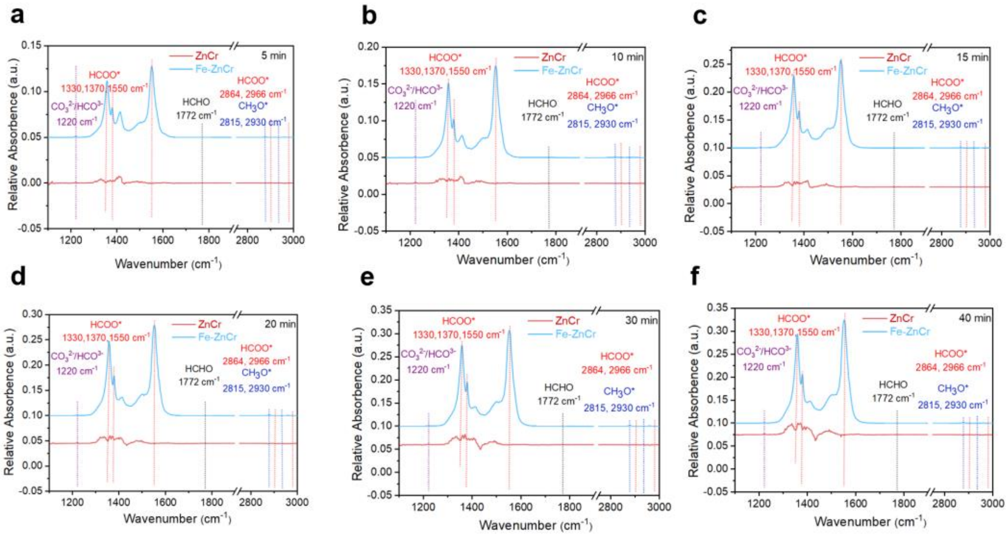

Supplementary Figure 13 | Detection of intermediates in CO hydrogen process. In-situ DRIFT spectra for the CO hydrogenation over $ZnCr_2O_4$ oxide 4.48% Fe doping in $ZnCr_2O_4$ oxide from (a) 5 min (b) 10 min (c) 15 min (d) 20 min (e) 30 min (f) 40 min

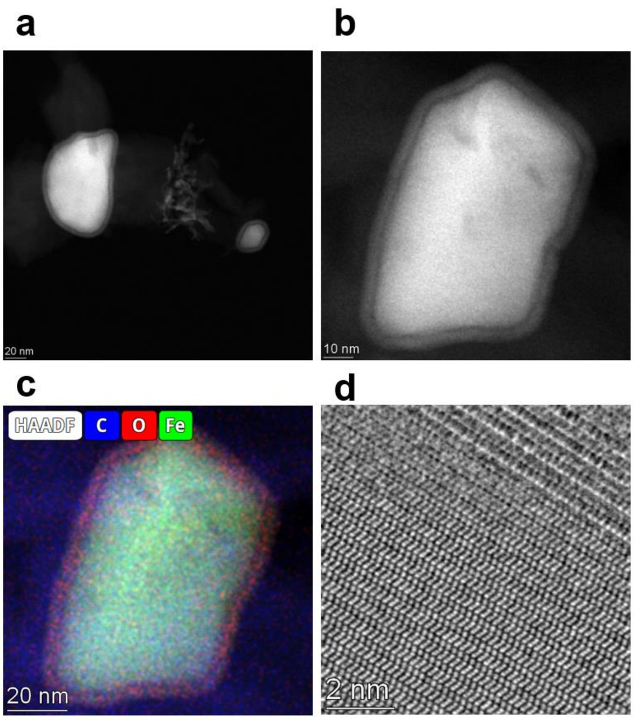

Supplementary Figure 14 | Structure characterization of spent enriched Fe samples. (a-b) HADDF-STEM images of spent Enriched Fe samples (c) EDS-mapping of Fe species in Enriched Fe samples (d) High resolution images of Enriched Fe (Fe_xC_y)

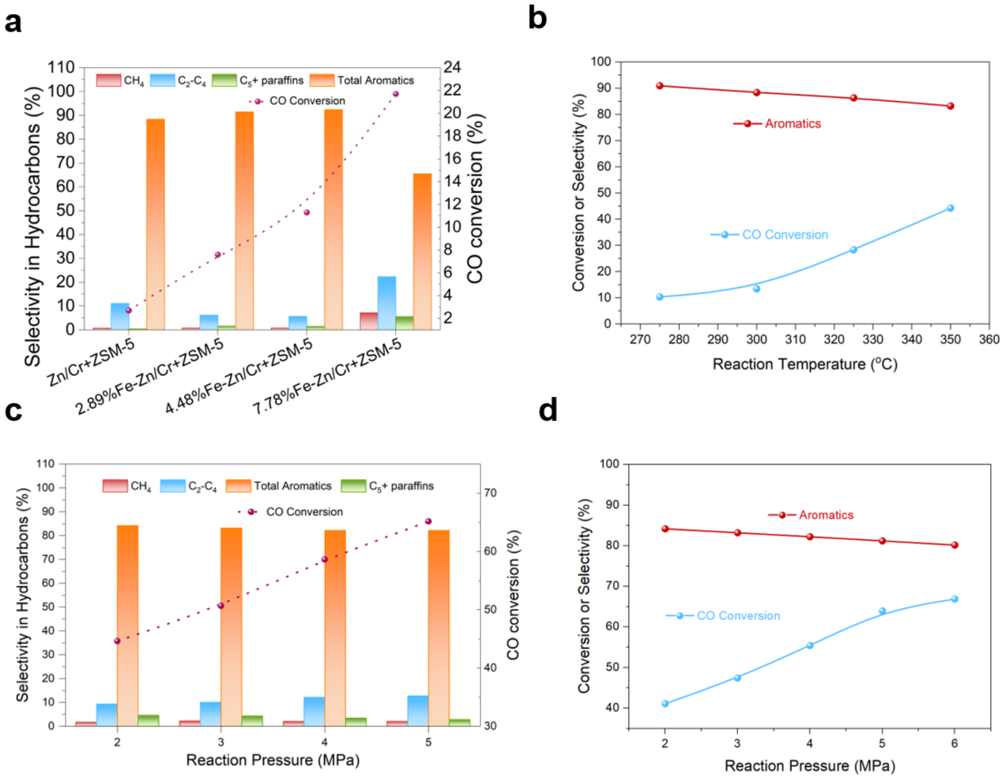

Supplementary Figure 15 | Catalytic evaluation of different Fe doping samples. (a) Catalyst evaluation of pristine $ZnCr_2O_4$ and Fe doping in $ZnCr_2O_4$ at a relative low temperature of 275 ^{o}C, 2.0MPa, 600ml/hr/g_{cat}, CO:H_2=1:1. (b) Catalyst evaluation of 4.48 wt% Fe doping samples with different reaction temperature from 275°C to 350°C, reaction conditions: 2.0MPa, 600 ml/hr/g_{cat} (c-d) Product distribution on 4.48 wt% Fe doping in $ZnCr_2O_4$ with different reaction pressure at 350 ^{o}C, 600ml/h/g_{cat}, CO:H_2= 1:1.

$ZnFe_2O_4$ was considered as an effective CO hydrogenation during F-T synthesis process and the catalyst evaluation of solo $ZnFe_2O_4$ and composite catalyst composed of $ZnFe_2O_4$ and H-ZSM-5 was exhibited in Figure S16. It can be speculated that $ZnFe_2O_4$ is not able to convert the intermediates like HCOH & CH_3O^* and when coupling with H-ZSM-5, the total aromatic selectivity is low although the CO conversion is very extremely high. Enrichment Fe is a compound effect bringing by the $ZnFe_2O_4$ which contains the local structure of Fe-O-Fe/adjacent Fe.

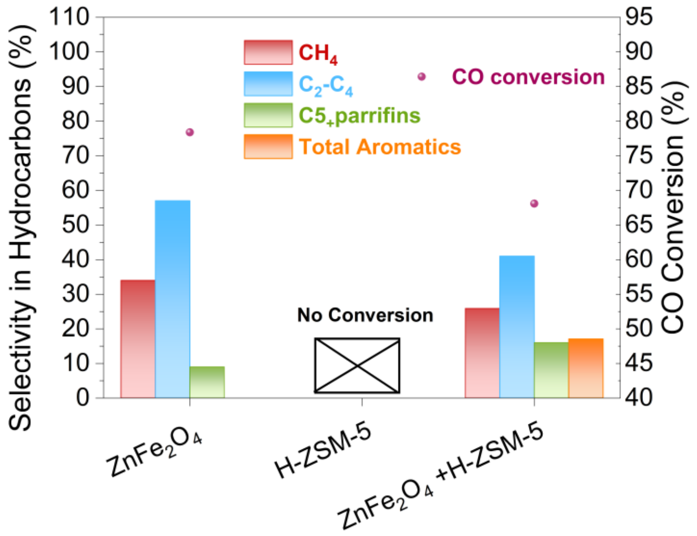

Supplementary Figure 16 | Catalytic evaluation over enriched Fe samples. CO hydrogenation over solo $ZnFe_2O_4$, H-ZSM-5 and composite $ZnFe_2O_4$ and H-ZSM-5 catalyst at reaction conditions of 350°C, 2.0MPa, 600ml/hr·gcat and CO/H_2=1:1

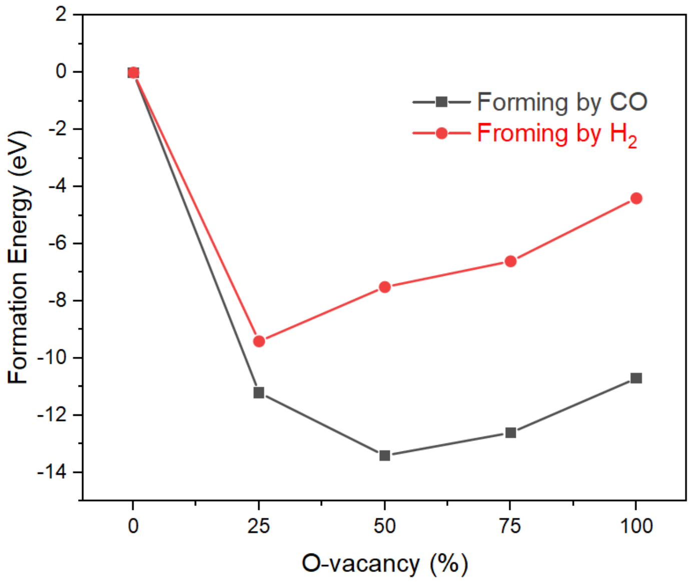

Supplementary Figure 17 | The forming energy of oxygen vacancy. DFT calculations of oxygen vacancy forming energy on Fe/$ZnCr_2O_4$[111] surface

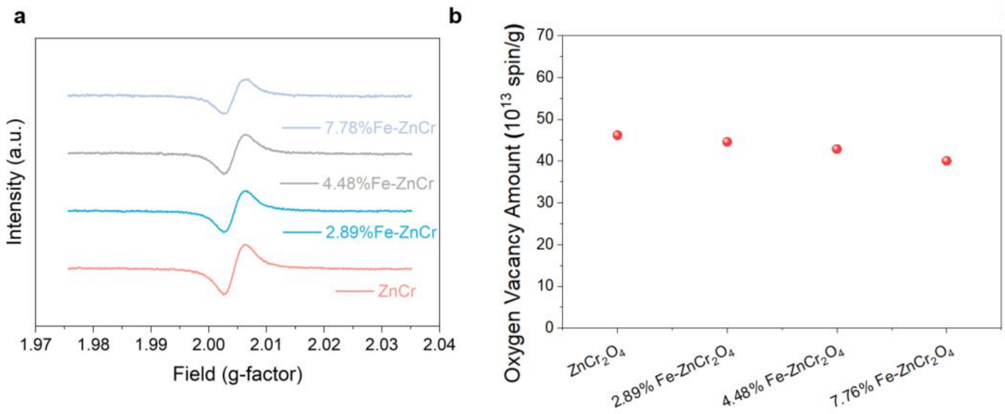

Supplementary Figure 18 | Determination of oxygen vacancy (a) EPR spectroscopy of different doping Fe samples and pristine $ZnCr_2O_4$ (b) Quantitative results of oxygen vacancies of different samples determined by EPR

To determine the CO activation ability of the four samples, in-situ CO Drifts was conducted at 173K and 1 atm pressure. The peak appears at 2043-2059 cm^{-1} is attributed to the vibrations of C-O in oxygen vacancies and 2157-2219 cm^{-1} peak is subscribed to the vibrations of C-O on the top metal sites[1, 2]. **Supplementary Figure 19** shows qualitative results of CO adsorption on oxygen vacancies versus top metal sites, which indicates that when doping Fe species in $ZnCr_2O_4$ matrix, the CO adsorption & activation on oxygen vacancies are strengthen although the amounts of oxygen vacancies are barely unchangeable. It means mono-dispersion Fe in 16d sites activates the surrounding oxygen vacancies thus increasing the CO activation ability, and this can be further demonstrated by the DFT calculations from the forming energy of oxygen vacancies.

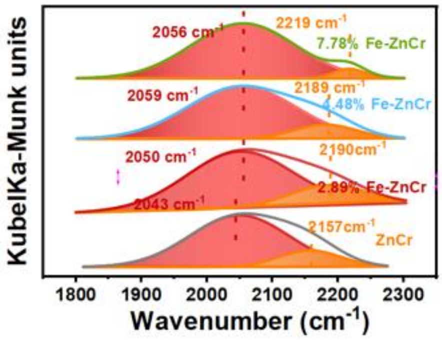

Supplementary Figure 19 | Determination of adsorption sites for CO. In-situ CO Drifts profile of different samples preformed at 173K

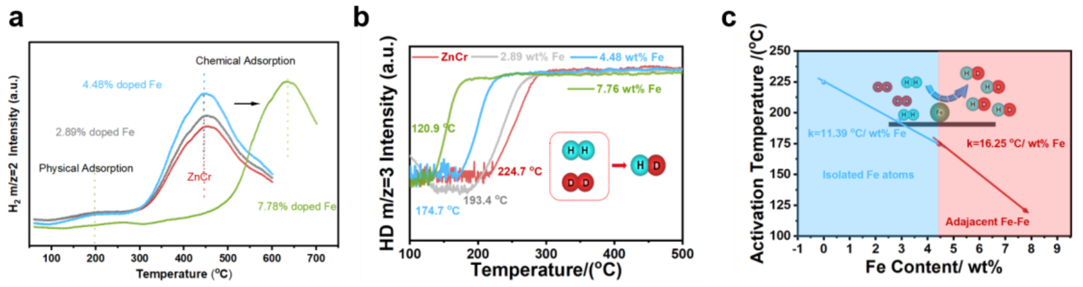

Supplementary Figure 20 | The H_2 activation ability over four samples. (a) H_2-TPD profile of different doping Fe samples versus pristine $ZnCr_2O_4$ (b) H_2 activation temperature versus Fe contents in $ZnCr_2O_4$ (c) H_2 activation Temperature versus Fe contents profile

H_2 adsorption behavior was detected by using H_2-TPD and **Supplementary Figure 20 (a)** shows the H_2 adsorption curves of different wt% Fe doping samples versus pristine $ZnCr_2O_4$. When Fe substitutes the Cr on 16d sites in isolation, H_2 desorption peak at 450°C is attributed to the chemical adsorption of H*[3]. It is obvious to see that H_2 adsorption is stronger than pristine $ZnCr_2O_4$ on 4.48 wt% Fe doping samples, indicating mono-dispersion Fe in 16d sites is able to adsorb and activate more H_2. Moreover, excess Fe changes the H_2 adsorption temperature from 450°C to 623°C due to the Fe-O-Fe structure, which gives an over-reinforced H_2 abilities[4]. Furthermore, to demonstrate the H_2 activation ability on Fe doping samples, H_2-D_2 exchange experiments were carried out. **Supplementary Figure 20 (b & c)** indicates that by increasing Fe doping amounts, the initial H_2 activation temperature was lowered from 224.7°C to 120.9°C. By analyzing the decrease of initial activation temperature versus Fe contents in $ZnCr_2O_4$ matrix, it can be concluded mono-dispersion Fe gives a 11.39°C/wt% Fe while enrichment Fe gives a 16.25°C/wt% Fe, which conforms our H_2 TPD profile well and it also demonstrates that enrichment Fe gives over-enhancement of H_2 activation. Mössbauer in **Supplementary Figure 1 (b)** shows the Γ increases from 0.36 to 1.27 caused by the enrichment of Fe, which has the potential to evolute the Fe_xC_y phases under the CO atmosphere. Moreover, DFT calculation was carried to compare H_2 dissociation ability on three top metal sites of Fe, Cr, Zn.

Due to the formation of Fe_5C_2 species, the C-C coupling is strengthened in enriched Fe samples. It better demonstrates the mono-dispersity in 2.89% and 4.48% Fe-ZnCr. Several studies have demonstrated that olefins also can be transformed into aromatics via cyclization, dehydration and hydrogen transfer to generate aromatics over Fe-based catalyst[5-7]. Therefore, in our reaction system, it is possible for some of the aromatics arise from olefins since the metal oxide catalyst can produce a small quantity of light olefins. It should be noted that C1 species are the main product on the solo metal oxide. Therefore, in our reaction system, it is deduced that C1 oxygenate before CH_4/CH_3OH are the key intermediates such as H_2CO.

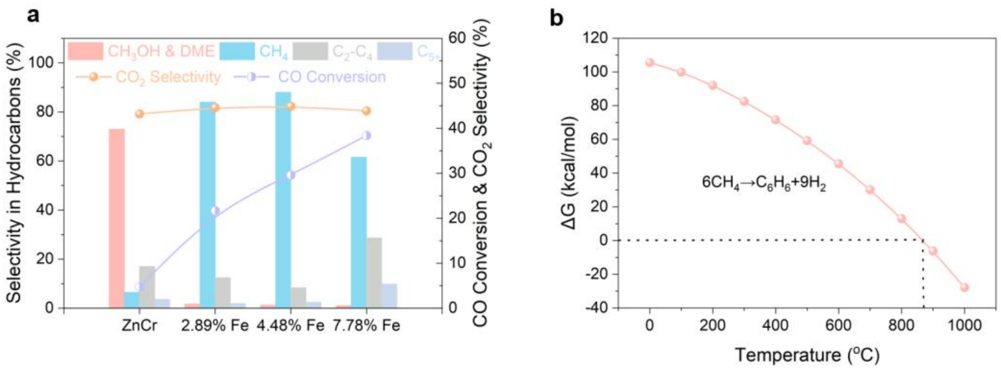

Supplementary Figure 21 | Catalytic evaluation of solo metal oxide. (a) CO hydrogenation over solo metal oxides at the condition of 350°C, 2.0 MPa, 600 ml/hr/g_{cat} (b) Thermodynamic analysis of CH_4 to aromatics

As shown in **Supplementary Figure 22**, in general, in a spinel system, metals in the 16d sites in the bulk should be coordinated with 6 oxygen atoms, indicating the O_h point group. When the 16d sites are near the surface, the top oxygen atom tends to combine with H_2/CO to form the H_2O or CO_2 under the reduction atmosphere. Therefore, in **Supplementary Table 1**, it is deduced that the Fe-O coordination can better demonstrate that Fe is monodispersed in the 16d sites of $ZnCr_2O_4$ with a coordination number at the range of 4.69-5.01.

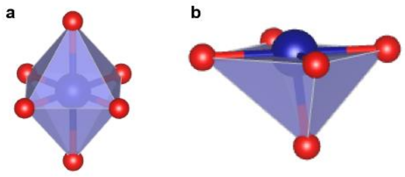

Supplementary Figure 22 | Atomic structural model of first shell coordination of Fe. The coordination conditions of 16d sites metal in spinel (a) bulk (b) surface

As shown in **Supplementary Figure 23**, to keep the GHSV at the 600 ml/hr/g_{cat}, we increase the flow rate of CO from 5 to 25 ml/min and the CO conversion keeps at the range of 11-13%, indicating that the reaction removes the mass transfer limitations.

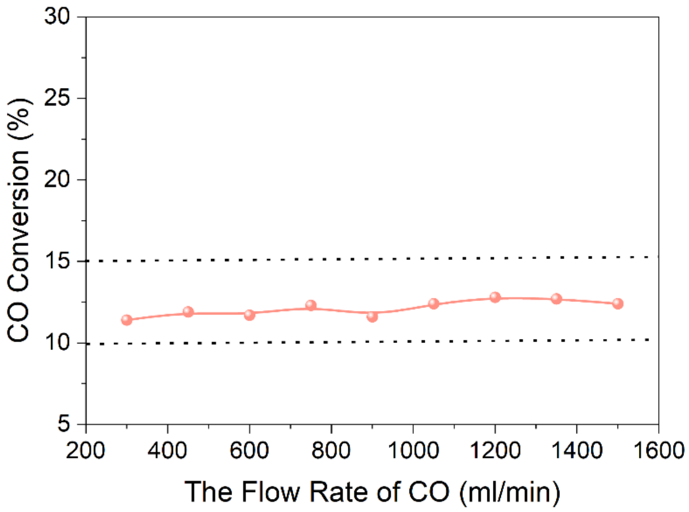

Supplementary Figure 23 | Remove of diffusion limitations for inter-particles. CO conversion over the flow rate of CO over $ZnCr_2O_4$ and H-ZSM-5 composite catalysts.

As shown in **Supplementary Figure 24**, the CO_2 selectivity of four samples is in the range of 44%-47%, which means the low cost of valuable H_2 based on the oxygen balance.

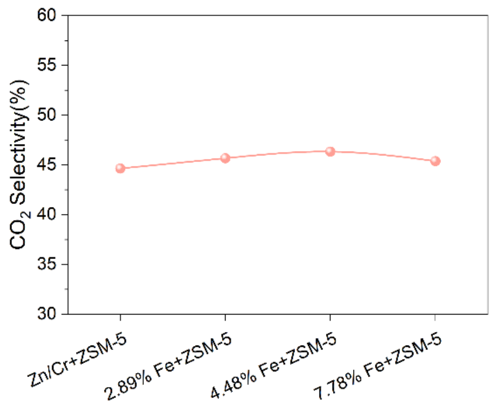

Supplementary Figure 24 | The CO_2 selectivity over pristine and Fe-doping catalysts. CO_2 selectivity of four metal oxide samples when combining with H-ZSM-5 at the conditions of 350°C, 2.0MPa and 600ml/hr/g_{cat}

As shown in **Supplementary Figure 25**, by checking the procedure, the aromatics included in the selectivity tabulation in Figure 2 are BTX (2.7%), trimethlybenzene (36.9%), tetramethylbenzene (54.2%), pentamethylbenzene (3.7%) and hexamethylbenzene (2.5%).

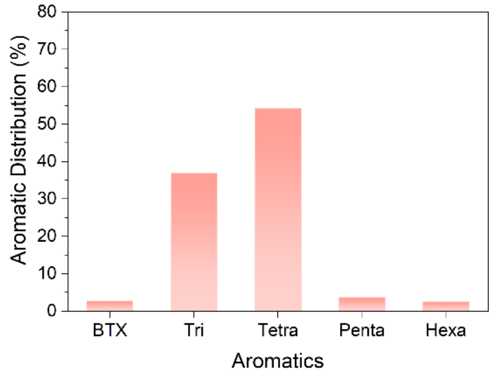

Supplementary Figure 25 | The aromatic distribution of monodispersed Fe-ZnCr+H-ZSM-5 catalysts. Aromatic distribution in 4.48 wt% Fe/ZnCr+H-ZSM-5 samples

Supplementary Table 1 EXAFS fitting parameters at the Fe K-edge of different Fe doping in $ZnCr_2O_4$ samples

Samples	Shell	CN	R (Å)	σ^2 (Å^2)	ΔE_0(eV)	R factor
2.89%	Fe-O	5.01	1.95	0.006	-3.86	0.0009
Fe/ZnCr	Fe-Cr	4.69	2.94	0.007	9.69	0.0023
4.48%	Fe-O	4.71	1.95	0.007	-3.30	0.0014
Fe/ZnCr	Fe-Cr	4.38	2.93	0.003	9.93	0.0005
7.78%	Fe-O	4.69	1.95	0.009	-4.57	0.0114
Fe/ZnCr	Fe-Cr & Fe-Fe	4.19	2.93 & 2.81	0.006	9.98	0.0212

Supplementary Table 2 Reaction energy of mono-dispersion Fe and $ZnFe_2O_4$ to the Fe_5C_2 species

Reaction	ΔG/eV
$ZnFe_2O_4+0.8CO+3.8H_2 \rightarrow 0.4Fe_5C_2+ZnO+3.8H_2O$	-3.17
$Fe/ZnCrO_x+0.8CO+0.8H_2 \rightarrow ZnCrO_x+0.4Fe_5C_2+0.8H_2O$	1.25

Supplementary Table 3 Fitting data for Mössbauer spectra of different doping Fe samples

	IS (mm/s)	QS (mm/s)	Γ(mm/s)	Area (%)	Valence-state
Doublet1	0.14	0.58	0.60	84.2	Fe^{III} low-spin
Doublet2	0.26	1.22	0.36	15.8	Fe^{III} high-spin
	IS (mm/s)	QS (mm/s)	Γ(mm/s)	Area (%)	Valence-state
Doublet1	0.16	0.68	0.58	78.1	Fe^{III} low-spin
Doublet2	0.27	1.30	0.39	21.9	Fe^{III} high-spin
	IS (mm/s)	QS (mm/s)	Γ(mm/s)	Area (%)	Valence-state
Doublet1	0.18	0.66	0.53	73.0	Fe^{III} low-spin
Doublet2	0.36	1.36	1.27	27.0	Fe^{III} high-spin

Supplementary Table 4 The catalytic activities of CO_x hydrogenation to aromatics over kinds of zeolite-supported metal catalysts.

Catalyst	H_2/CO ratio	T(°C)	P(MPa)	CO_x conversion (%)	Aromatic Selectivity (%)	GHSV (ml/hr/gcat)	References
Na/Fe+H-ZSM-5	3.0	340	2.0	34.2	47.6	4800	[8]
FeMn@MZ5	1.0	320	2.0	51.9	47.1	3000	[9]
FeZnNa+H-ZSM-5	2.7	340	2.0	88.8	50.6	8800	[10]
Fe_5C_2+ZnCr+H-ZSM-5	2.0	350	3.0	46.4	73	1500	[11]
ZnO-X-H-ZSM-5	2.0	350	3.0	22.5	74.3	1000	[12]
FeNaMg+Ni-H-ZSM-5	2.0	350	2.0	95.1	53.6	1200	[13]
ZnO-ZrO2-H-ZSM-5	3.0	320	4.0	14.1	73.0	900	[14]
MnCr-Z5-	2.0	350	3.0	11.2	59.0	900	[15]

Si4							
ZrO2/H-ZSM-5	2.0	350	3.0	14.7	64.4	600	[16]
Ce0.2Zr0.8O2-Z-5-Si4	2.0	350	3.0	12.2	84.1	900	[17]
Monodispersed Fe-ZnCr+H-ZSM-5 (This Work)	1.0	350	4.0	64.3	82.8	600	This Work

Supplementary Table 5 The amounts of active sites and TOF values

Catalysts	Active sites (μmol· g^{-1}) [a]	TOF (mol s^{-1})
Pristine ZnCr	12.87[a]	0.138[a]
2.89% Fe-ZnCr	14.76[a]	0.383[a]
4.48% Fe-ZnCr	15.48[a]	0.484[a]
7.78% Fe-ZnCr	15.96[a]	0.560[a]

a. Determined by chemisorption of CO and H_2

Supplementary References:

1. Guan, Q. et al. Bimetallic monolayer catalyst breaks the activity–selectivity trade-off on metal particle size for efficient chemoselective hydrogenations. *Nat. Catal.* **4**, 840-849 (2021).
2. Hu, J. et al. Sulfur vacancy-rich MoS2 as a catalyst for the hydrogenation of CO2 to methanol. *Nat. Catal.* **4**, 242-250 (2021).
3. Cheng, K. et al. Bifunctional Catalysts for One-Step Conversion of Syngas into Aromatics with Excellent Selectivity and Stability. *Chem* **3**, 334-347 (2017).
4. Sun, X.-X., Du, J., Tan, J.-J. & Zhan, S.-Z. A mono-oxo-bridged binuclear iron (iii) complex with a Fe–O–Fe angle of 180.0° and its catalytic activity for hydrogen evolution. *New J. Chem.* (2022).
5. Zhao, B. et al. Direct Transformation of Syngas to Aromatics over Na-Zn-Fe 5 C 2 and Hierarchical HZSM-5 Tandem Catalysts. *Chem* **3**, 323-333 (2017).
6. Xu, Y. et al. Selective conversion of syngas to aromatics over Fe3O4@ MnO2 and hollow HZSM-5 bifunctional catalysts. *ACS Catal.* **9**, 5147-5156 (2019).
7. Xu, Y., Liu, D. & Liu, X. Conversion of syngas toward aromatics over hybrid Fe-based Fischer-Tropsch catalysts and HZSM-5 zeolites. *Appl. Catal. A: Gen.* **552**, 168-183 (2018).
8. Yang, X. et al. Exploring the reaction paths in the consecutive Fe-based FT catalyst–zeolite process for syngas conversion. *ACS Catal.* **10**, 3797-3806 (2020).
9. Xu, Y. et al. Yolk@ Shell FeMn@ Hollow HZSM-5 nanoreactor for directly converting syngas to aromatics. *ACS Catal.* **11**, 4476-4485 (2021).
10. Zhao, B. et al. Direct transformation of syngas to aromatics over Na-Zn-Fe5C2 and hierarchical HZSM-5 tandem catalysts. *Chem* **3**, 323-333 (2017).
11. Fu, Y., Ni, Y., Chen, Z., Zhu, W. & Liu, Z. Achieving high conversion of syngas to aromatics. *J. Energy Chem.* **66**, 597-602 (2022).
12. Fu, Y., Ni, Y., Zhu, W. & Liu, Z. Enhancing syngas-to-aromatics performance of ZnO&H-ZSM-5 composite catalyst via Mn modulation. *J. Catal.* **383**, 97-102 (2020).
13. Sun, Q., Wang, N. & Yu, J. Advances in Catalytic Applications of Zeolite-Supported Metal Catalysts. *Adv. Mater.* **33**, 2104442 (2021).
14. Zhou, W. et al. Direct conversion of syngas into aromatics over a bifunctional catalyst: inhibiting net CO 2 release. *Chem. Commun.* **56**, 5239-5242 (2020).
15. Miao, D. et al. Selective synthesis of benzene, toluene, and xylenes from syngas. *ACS Catal.* **10**, 7389-7397 (2020).
16. Zhou, W. et al. Selective Conversion of Syngas to Aromatics over a Mo− ZrO2/H-ZSM-5 Bifunctional Catalyst. *ChemCatChem* **11**, 1681-1688 (2019).
17. Huang, Z. et al. Ceria-Zirconia/Zeolite Bifunctional Catalyst for Highly Selective Conversion of Syngas into Aromatics. *ChemCatChem* **10**, 4519-4524 (2018).
